# GSH/pH-Responsive Chitosan–PLA Hybrid Nanosystems for Targeted Ledipasvir Delivery to HepG2 Cells: Controlled Release, Improved Selectivity, DNA Interaction, Electrochemical and Stopped-Flow Kinetics Analyses

**DOI:** 10.3390/ijms26136070

**Published:** 2025-06-24

**Authors:** Ahmed M. Albasiony, Amr M. Beltagi, Mohamed M. Ibrahim, Shaban Y. Shaban, Rudi van Eldik

**Affiliations:** 1 Analytical and Bioinorganic Chemistry Departments, Faculty of Science, Kafrelsheikh University, Kafrelsheikh 33516, Egypt; ahmed_albasiony@sci.kfs.edu.eg (A.M.A.); ambeltagi@hotmail.com (A.M.B.); 2 Department of Chemistry, College of Science, Taif University, P.O. Box 11099, Taif 21944, Saudi Arabia; ibrahim@tu.edu.sa; 3 Departments of Chemistry and Pharmacy, University of Erlangen-Nuremberg, 91058 Erlangen, Germany; 4 Faculty of Chemistry, Nicolaus Copernicus University in Torun, 87100 Torun, Poland

**Keywords:** chitosan nanoparticles, pH-responsive, GSH-responsive, targeted drug delivery, ledipasvir repurposing, therapeutic index

## Abstract

This study aimed to design dual-responsive chitosan–polylactic acid nanosystems (PLA@CS NPs) for controlled and targeted ledipasvir (LED) delivery to HepG2 liver cancer cells, thereby reducing the systemic toxicity and improving the therapeutic selectivity. Two formulations were developed utilizing ionotropic gelation and *w*/*o*/*w* emulsion techniques: LED@CS NPs with a size of 143 nm, a zeta potential of +43.5 mV, and a loading capacity of 44.1%, and LED-PLA@CS NPs measuring 394 nm, with a zeta potential of +33.3 mV and a loading capacity of 89.3%, with the latter demonstrating significant drug payload capacity. Since most drugs work through interaction with DNA, the in vitro affinity of DNA to LED and its encapsulated forms was assessed using stopped-flow and other approaches. They bind through multi-modal electrostatic and intercalative modes via two reversible processes: a fast complexation followed by a slow isomerization. The overall binding activation parameters for LED (cordination affinity, *K*_a_ = 128.4 M^−1^, *K*_d_ = 7.8 × 10^−3^ M, Δ*G* = −12.02 kJ mol^−1^), LED@CS NPs (*K*_a_ = 2131 M^−1^, *K*_d_ = 0.47 × 10^−3^ M, Δ*G* = −18.98 kJ mol^−1^) and LED-PLA@CS NPs (*K*_a_ = 22026 M^−1^, *K*_d_ = 0.045 × 10^−3^ M, Δ*G* = −24.79 kJ mol^−1^) were obtained with a reactivity ratio of 1/16/170 (LED/LED@CS NPs/LED-PLA@CS NPs). This indicates that encapsulation enhanced the interaction between the DNA and the LED-loaded nanoparticle systems, without changing the mechanism, and formed thermodynamically stable complexes. The drug release kinetics were assessed under tumor-mimetic conditions (pH 5.5, 10 mM GSH) and physiological settings (pH 7.4, 2 μM GSH). The LED@CS NPs and LED-PLA@CS NPs exhibited drug release rates of 88.0% and 73%, respectively, under dual stimuli over 50 h, exceeding the release rates observed under physiological conditions, which were 58% and 54%, thereby indicating that the LED@CS NPs and LED-PLA@CS NPs systems specifically target malignant tissue. Release regulated by Fickian diffusion facilitates tumor-specific payload delivery. Although encapsulation did not enhance the immediate cytotoxicity compared to free LED, as demonstrated by an in vitro cytotoxicity in HepG2 cancer cell lines, it significantly enhanced the therapeutic index (2.1-fold for LED-PLA@CS NPs) by protecting non-cancerous cells. Additionally, the nanoparticles demonstrated broad-spectrum antibacterial effects, suggesting efficacy in the prevention of chemotherapy-related infections. The dual-responsive LED-PLA@CS NPs allowed controlled tumor-targeted LED delivery with better selectivity and lower off-target toxicity, making LED-PLA@CS NPs interesting candidates for repurposing HCV treatments into safer cancer nanomedicines. Furthermore, this thorough analysis offers useful reference information for comprehending the interaction between drugs and DNA.

## 1. Introduction

Cancer is a major global health concern, with over eight million deaths from the disease each year and predictions that the number will rise to 11–12 million by 2030 [1,2]. The high death rate of cancer endures despite advances in our understanding of its biology, primarily because of the shortcomings of the available treatment options. The main features of cancer mortality and treatment difficulties are described in the sections that follow. With almost 10 million deaths worldwide, lung cancer ranked as the top cause of cancer-related mortality in 2020 [3]. Higher rates of cancer mortality are found in wealthy places; however, many low- and middle-income nations also experience significant mortality from the disease [3]. Radiation and chemotherapy are two examples of current treatments that frequently lack selectivity, harming both malignant cells and healthy tissues [2]. Because of their non-discriminatory action on rapidly dividing cells, like those in soft tissues and hair, chemotherapy medicines, notwithstanding their effectiveness, are associated with serious adverse effects [4,5].

The need for more research and innovation in cancer care is underscored by the persistent difficulties involved in obtaining the selective targeting of medicines, despite the noteworthy achievements in cancer treatment. To obtain the greatest outcomes, a highly specialized drug delivery system that can release the chemotherapeutic agents in a controlled manner must be used in conjunction with the right medicine dosage delivery and therapy duration. The primary concern for such a drug delivery system is delivering the drug at safe and optimal levels compared to currently used treatment procedures [6,7,8]. Targeting tumors has grown in popularity as a way to reach tumor sites without penetrating healthy tissue’s interstitial space. Drug delivery methods based on nanoscale technology are currently receiving a lot of attention from researchers in an effort to target cancer cells [9,10,11,12,13,14,15,16]. Stimuli-responsive lipid-based drug delivery systems, prodrugs, and nanocarriers have attracted the most attention among these strategies. Improved selectivity, biocompatibility, sensitivity to the cancer microenvironment, and clinical acceptability are some of these systems’ salient features. They also provide extra benefits, including flexibility in selecting formulation components and scalability. Stimuli-responsive carriers can improve the medication distribution throughout the tumor volume, promote cellular binding and internalization, or enable faster or triggered drug release at the tumor site [17]. Recent developments have produced a number of stimuli-responsive drug delivery systems that can greatly increase tumor necrosis. Systems based on biocompatible excipients are also used for tumor-specific localization, covert characteristics, and effective bodily removal. For theranostic uses of anti-cancer medicines, these cutting-edge drug delivery platforms can efficiently support gene therapy, chemotherapy, or a combination of the two [18]. Numerous innovative formulations of these delivery systems use polymers [19], hydrogels [20], and nanoparticles [21].

A natural polymer with biocompatibility and biodegradability, chitosan has demonstrated potential as a medication delivery technology based on nanoparticles. It is a great option because of its mucoadhesive qualities, capacity to enhance intestinal absorption, and ability to shield encapsulated medications from enzymatic degradation [22,23]. According to research, chitosan nanoparticles increase oral bioavailability by facilitating regulated drug release and prolonging gastrointestinal retention [24]. Moreover, chitosan’s capacity to create hydrogels improves its usefulness in medication delivery systems, enabling regulated release and increased therapeutic effectiveness [25]. On the other hand, biodegradable polyester made from renewable resources like sugar cane or maize starch is known as polylactic acid (PLA). Polylactic acid (PLA) was selected for its biocompatibility, biodegradability, and ability to form stable nanoparticles with sustained-release properties [26]. While concerns exist about microplastics formation from PLA’s degradation in vivo, its breakdown into lactic acid, a naturally occurring metabolite, minimizes the long-term toxicity risks [26]. Because of its capacity to replicate the extracellular matrix (ECM), PLA has advantageous mechanical qualities and is frequently utilized in the medical industry. Because PLA is so adaptable, its chemical and physical characteristics can be changed to create scaffolds that are specifically suited to a given medicinal application [26].

**Scheme 1:**
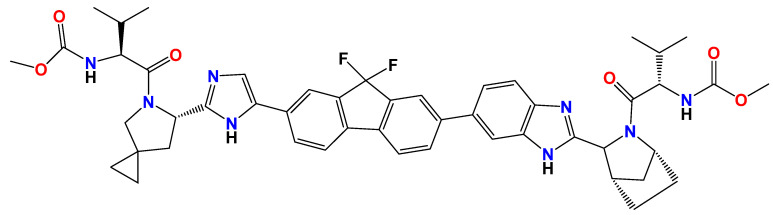
Chemical structure of ledipasvir.

Due to the biodegradability of PLA and the mucoadhesive qualities of chitosan, which enhance cellular uptake and retention, hybrid nanoparticles based on PLA and chitosan have become potential carriers [27,28]. By taking advantage of the acidity of the tumor microenvironment (pH 6.5–6.8) and the increased intracellular GSH levels (2–10 mM) in cancer cells, these systems become even more sophisticated when they are designed with dual pH/glutathione (GSH) responsiveness [8,28]. Though mainly known as an inhibitor of hepatitis C NS5A [29], ledipasvir (Scheme 1) exhibits untapped promise in oncology through its postulated DNA interaction pathways. Advanced delivery techniques are required due to its non-specific biodistribution and limited aqueous solubility [29].

The drug distribution is optimized when chitosan and polylactic acid (PLA) are combined because it improves the structural stability and permits variable breakdown rates. The hydrophobic properties of PLA enhance the stability of the nanoparticles, while the positive charge of chitosan improves the cellular absorption in this dual-polymer system. Such a method could allow for tailored distribution of ledipasvir to liver cells while preventing precipitation under physiological conditions. Improved liver retention has already been shown in preclinical animals employing liposomes coated with galactosylated chitosan [30,31]. Developments in chitosan–PLA hybrid systems demonstrate how well they can encapsulate medications that are poorly soluble. For example, electrospun membranes are biocompatible and have antibacterial characteristics [32]. These developments are consistent with the wider uses of chitosan-based carriers to improve the bioavailability of difficult-to-treat medications such as phytochemicals and antivirals [22,23]. This strategy tackles important formulation concerns for ledipasvir while providing flexible solutions for both juvenile and elderly patients by fusing the sticky qualities of chitosan with PLA’s sustained-release capabilities [33].

In this study, we synthesized LED@CS NPs and LED-PLA@CS NPs, wherein the chemotherapeutic agent LED was encapsulated within CS NPs and PLA@CS NPs with the objective of developing dual pH- and glutathione-responsive nanosystems for the controlled delivery of ledipasvir to HepG2 liver cancer cells. Using cyclic voltammetry, scanning electron microscopy (SEM), Fourier transform infrared spectroscopy (FT-IR), the zeta potential, and the particle size, the chemical and physical properties of the LED@CS NPs and LED-PLA@CS NPs were characterized. For many drugs currently in use (such as cisplatin, carboplatin, and oxaliplatin) that have received FDA approval, DNA is the main pharmacological target [34]. It was proposed that the ability they had to bind DNA was most likely responsible for their cytotoxic effects [35]. We employed a model of double-helical genomic DNA (calf thymus, ct-DNA) to investigate the in vitro binding of our materials to DNA using cyclic voltammetry, differential scanning calorimetry (DSC) measurements, and UV–Vis spectroscopy. Because the DNA–drug affinity is thought to be fast occurring, we performed a kinetic investigation of the interaction process using a stopped-flow approach. This technique clarifies the interaction process by enabling the determination of the kinetic characteristics of the phases that generate intermediate species. In-depth evaluation and further explanation of the drug release kinetics of LED@CS NPs and LED-PLA@CS NPs, which ensure ideal dosage timings, as well as their cytotoxicity against the diseased cell line, the hepatocellular carcinoma (HepG2) cell line, in comparison to non-cancerous normal cells, WI-38 cells, were provided. Chemotherapy-induced immunosuppression increases the infection risk, so antimicrobial co-therapies improve survival compared to chemotherapy alone [35,36]. Therefore, six bacterial strains (*B. subtilis*, *K. pneumoniae*, *S. aureus*, *E. coli*, *S. typhi*, *E. faecalis*) were used to test the antibacterial effects of free and encapsulated ledipasvir in vitro using the agar diffusion method.

## 2. Results and Discussion

### 2.1. Preparation and Characterization

CS and PLA@CS NPs loaded with LED were produced using non-covalent interactions, such as the hydrogen bond between the positively charged amino groups in CS and the hydroxyl groups of LED and PLA, with sodium tripolyphosphate (TPP) as the emulsifier, using the ionic gelation technique [37,38].

#### 2.1.1. FT-IR Analysis

**Figure 1:**
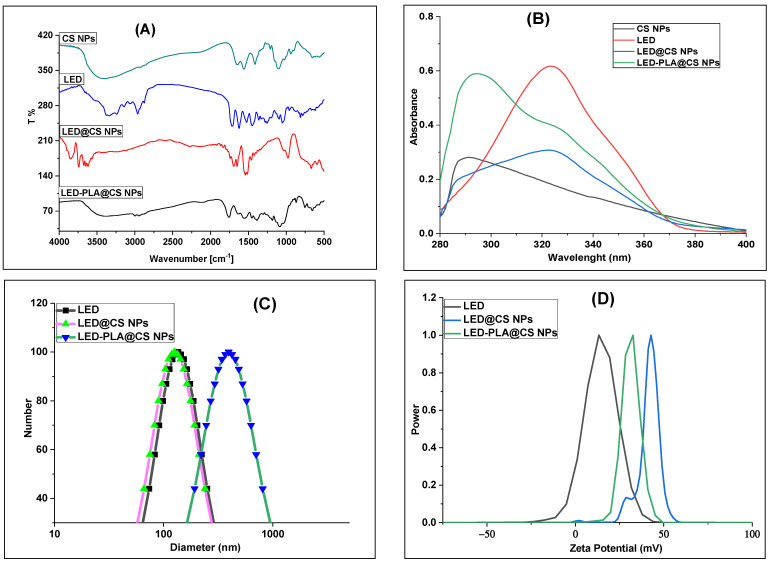
(**A**) FTIR spectra, (**B**) UV–vis spectra, (**C**) DLS of LED, LED@CS NPs and LED-PLA@CS NPs, and (**D**) zeta potentials.

The chemical interactions between LED and the encapsulated formulations were examined using FTIR spectroscopy (Figure 1A). A broad absorption band at about 3412 cm^−1^ was visible in the FTIR spectra of CS NPs, which is suggestive of overlapping O–H and N–H stretching vibrations. The C=O stretching of the amide I band was identified by a peak at 1648 cm^−1^, while the N–H bending vibration of the amide II band was identified by a peak at 1555 cm^−1^. The C–N stretching vibrations were characterized by bands at 1149 cm^−1^ and 1103 cm^−1^ [39,40,41]. N–H stretching vibration was identified as the cause of the steep peak in the LED spectrum at 3339 cm^−1^, whereas C–H stretching vibration was identified as the cause of the peak at 2963 cm^−1^. Due to the C=O stretching vibration, a significant absorption band was seen at 1704 cm^−1^. C=O stretching was also responsible for another peak at 1617 cm^−1^. The O–H stretching vibrations were indicated by a wide band in the LED@CS NPs spectra at 3434 cm^−1^. C=O stretching vibration was identified as the reason for the peak at 1739 cm^−1^ and another C=O stretching peak at 1693 cm^−1^. At 1537 cm^−1^, N–H bending vibration was detected, and C–O–C stretching vibrations were identified as the source of a peak at 1033 cm^−1^. It is possible that free O–H stretching triggered the peak at 3618 cm^−1^. The successful encapsulation of the LED in the CS NPs was confirmed by the existence of distinctive peaks from both the LED and CS NPs in the LED@CS NPs spectrum. O–H and N–H stretching, C–H stretching, and C=O stretching were among the important peaks. The overlap of the O–H and N–H stretching vibrations was identified as the cause of the broad band at 3391 cm^−1^ in the spectrum of LED-PLA@CS NPs. Using C–H stretching, peaks at 2996 cm^−1^ and 2946 cm^−1^ were identified. C=O stretching vibration was responsible for a prominent peak at 1756 cm^−1^, whilst C–H bending vibrations were responsible for the peaks at 1454 cm^−1^ and 1387 cm^−1^. The peak at 1087 cm^−1^ was attributed to C–O stretching vibration, whereas N–H bending was seen at 1553 cm^−1^. The successful creation of LED-PLA@CS NPs was validated by the existence of peaks in the composite’s spectra that are typical of PLA, LED, and CS NPs. In addition to the LED and CS NP peaks, other important peaks were those linked to C–O–C reaching from PLA. The effective synthesis of LED-PLA@CS NPs was further corroborated by the overlap of the O–H and N–H stretching vibrations at about 3391 cm^−1^, as well as the peaks at 1756 cm^−1^ (C=O stretching), 1454 cm^−1^ (C–H bending), and 1087 cm^−1^ (C–O stretching).

#### 2.1.2. UV–Vis Analysis

Drug loading and nanoparticle production are confirmed by the UV–vis spectra of the CS NPs, LED, LED@CS NPs, and LED-PLA@CS NPs, which are shown in Figure 1B. The broad 290–300 nm absorption band seen in the CS NPs is characteristic of chitosan. The conjugated aromatic system of pure LED is reflected in its absorption of approximately 0.61 at 325–330 nm [42]. A red-shifted peak with decreased intensity (~0.32) at 330–340 nm is seen in the LED@CS NPs, suggesting that LEDs are encapsulated by hydrogen bonds or non-covalent interactions. The bimodal profile of the LED-PLA@CS NPs is characterized by a shoulder at 330–340 nm (encapsulated LED) and significant absorption at 290–300 nm (ascribed to the PLA coating). The intensity variations and hypsochromic shift point to electrical interactions between the polymer matrix and the LED. By altering the optical characteristics and enhancing the stability, the PLA layer probably improves the photoprotection [43].

#### 2.1.3. Particle Sizes and Surface Charges

When evaluating the stability and properties of nanoparticles (NPs) for drug delivery, the mean particle size, polydispersity index (PDI), and zeta potential are crucial factors. To ascertain the precise size distribution of LED, LED@CS NPs, and LED-PLA@CS NPs, dynamic light scattering (DLS) was employed (Figure 1C,D and Table 1). LED had an average size of 152.4 nm, and after encapsulation in CS NPs, the size decreased to 142.5 nm. When polylactic acid was added, the size grew to 393.7 nm (LED-PLA@CS NPs), indicating that that the PLA coating of the LED@CS NPs was successful. The hydrodynamic diameters they displayed are thought to be ideal for maximizing the increased permeability and retention (EPR) effect [44]. Nanoparticles target tumors primarily through the enhanced permeability and retention (EPR) effect, leveraging their size (143–394 nm) to exploit the leaky vasculature and poor lymphatic drainage of tumors [44]. The positive zeta potential of LED@CS NPs (+43.51 mV) and LED-PLA@CS NPs (+33.27 mV) enhances cellular uptake via electrostatic interactions with negatively charged cancer cell membranes. However, the reliability of the EPR effect varies across tumor types and stages due to the heterogeneity in the vascular permeability and the tumor microenvironment [17]. Future studies should validate EPR-mediated targeting in vivo and explore active targeting strategies, such as ligand conjugation, to enhance the specificity. The sample’s particle size distribution is determined by the PDI. A population that is generally homogeneous is typically indicated by PDI values less than 0.3, whereas a system that is more polydisperse is suggested by values greater than 0.3. For LED, LED@CS NPs, and LED-PLA@CS NPs, the corresponding PDI values were 0.277, 0.304, and 0.389. Variations in the coating thickness or aggregation may be the cause of the increase in the PDI following PLA coating, which indicates wider size dispersion. The zeta potential predicts the stability of the colloidal system and quantifies the surface charge of the nanoparticles [45]. For LED, the zeta potential was +9.44 mV. The zeta potential rose to +43.51 mV following the synthesis of LED@CS NPs, suggesting enhanced colloidal stability brought on by the positive charge of chitosan. The zeta potential for the LED-PLA@CS NPs was +33.27 mV. The neutral or slightly negative charge of PLA, which lowers the total positive charge when coated on the LED@CS NPs, may be the cause of this drop as compared to LED@CS NPs.

#### 2.1.4. X-Ray Diffraction Analysis

**Figure 2:**
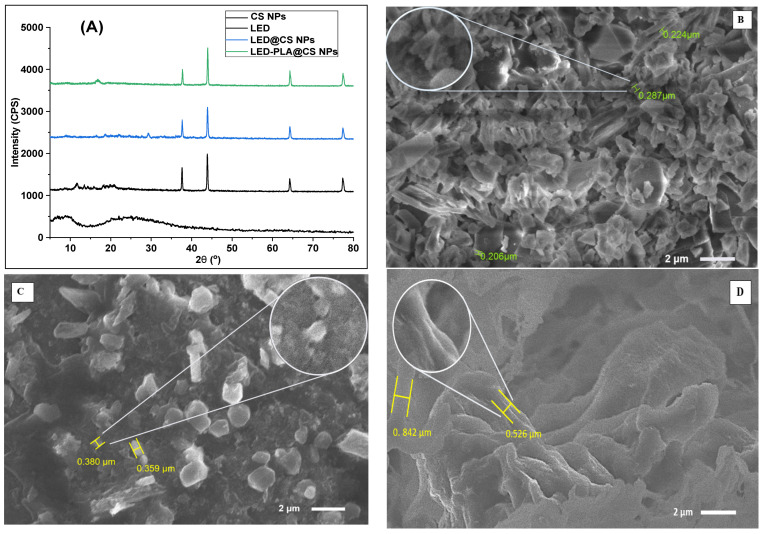
(**A**) XRD diffractograms of CS NPs, LED and its encapsulated forms; and SEM image of (**B**) LED, (**C**) LED@CS NPs and (**D**) LED-PLA@CS NPs.

To examine their structural characteristics, the XRD patterns of CS NPs, LED, LED@CS NPs, and LED-PLA@CS NPs were examined (Figure 2A). The amorphous nature of CS NPs is indicated by the large peak in the XRD pattern, which spans from 9 to 25 degrees 2θ. The large hump in this halo diffraction pattern represents the CS NPs’ mostly amorphous nature [46]. Sharp peaks at roughly 11.6°, 13.5°, 37.7°, 43.9°, 64.3°, and 77.4° 2θ are visible in the LED’s XRD pattern, indicating that it is crystalline. The effective encapsulation of LED in CS NPs is shown by the formation of a single new band at 2θ = 29.3° and the reduction in intensity or full fading of all the LED peaks following conjugation between CS NPs and LED in LED@CS NPs. It is also noteworthy that the CS NPs’ peaks experience deformation and intensity reduction, which is caused by the steric effect and hydrogen bonds between CS and LED, which significantly disrupt the orderly packing of polymer chains [40]. When PLA, LED, and CS were combined to create LED-PLA@CS NPs, the diffractogram revealed a new PLA-typical peak at 2θ = 16.8°, which is comparable with previous research [47,48]. This confirms the existence of PLA in the composite and aligns with the FTIR data. The phase crystallinity of LED did not alter, however, indicating that the synthesis did not eliminate the characteristics of LED’s distinctive structure. The medication maintains its crystalline character after encapsulation, as seen by the existence of LED peaks in the formulations for LED@CS NPs and LED-PLA@CS NPs. The lower concentration of the drug in the composite materials or the disruption of the drug’s crystal lattice during the encapsulation process may be the cause of the decreased intensity of the LED peaks in the encapsulated formulations as compared to the pure drug.

#### 2.1.5. Surface Morphological Analysis

The SEM pictures shown in Figure 2B–D offer important new information about the morphological properties of LED, both in its unadulterated state and when it is enclosed in nanoparticle formulations, emphasizing how these properties may affect the pharmacokinetics and therapeutic efficacy. The crystalline structure of pure LED is uneven, with rough surfaces, sharp edges, and diverse particle sizes (206–287 nm). Because of its low surface area-to-volume ratio, this shape, which is common in unprocessed medicines, restricts the bioavailability and dissolving rates. When LED is encapsulated in chitosan nanoparticles (CS NPs), its shape is drastically changed, producing more homogenous, spherical particles (356–380 nm) with smoother surfaces. These characteristics point to effective encapsulation inside the chitosan matrix, which improves the medication distribution and circulation time. The mucoadhesive and permeation-enhancing properties of chitosan facilitate regulated medication release kinetics and increase the oral bioavailability [49]. Additional polylactic acid (PLA) modification results in a layered morphology with sheet-like patterns and bigger particle sizes (565–842 nm). This probably shows that the PLA coating on top of the CS NPs created a complex structure that facilitates long-term drug release [50]. A multi-layered construction for extended-release characteristics, which lowers the dosage frequency and enhances patient compliance, can be indicated by the bigger size. The switch from crystalline LED (Figure 2B) to nanoparticle formulations (Figure 2C,D) indicates a calculated strategy for resolving issues with hydrophobic medicines’ bioavailability. While the PLA-modified formulation (Figure 2D) adds extended-release capabilities through biodegradable polymer breakdown, the homogeneity of the CS NPs (Figure 2C) facilitates regulated release. These morphological changes are essential for improving LED nano-formulations′ therapeutic potential.

#### 2.1.6. Electrochemical Characterization

**Figure 3:**
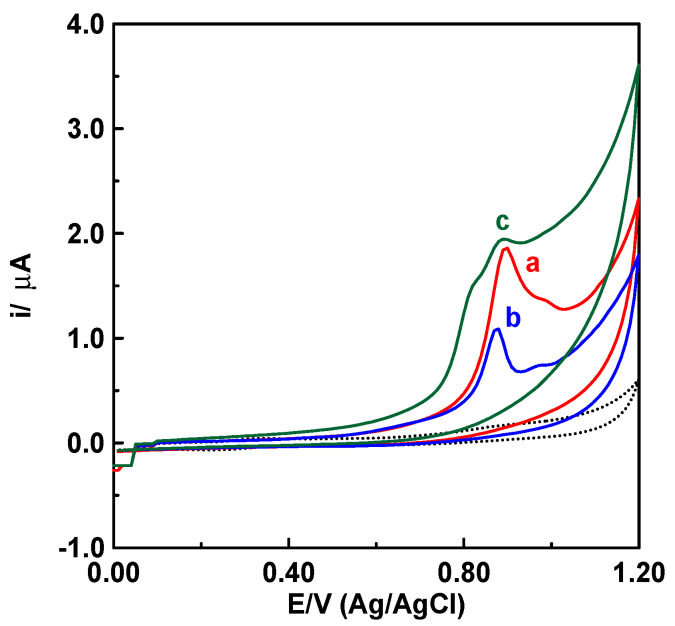
Cyclic voltammograms of 100 µM of (**a**) LED, (**b**) LED@CS NPs and (**c**) LED−PLA@CSNPs in acetate buffer solution at pH 4.8, applying a scan rate of 100 mV/s at a carbon paste electrode (CPE). The dashed curve represents the blank solution.

LED and its nano-formulations were also characterized using cyclic voltammetry. Figure 3 displays the cyclic voltammograms of 100 µM of LED and its nano-formulations. With a small peak shoulder at 0.990 V, the LED′s voltammogram displayed a single, clear irreversible anodic peak at 0.896 V. The four -NH groups in the drug moiety are oxidized by the 4e^−^/4H^+^ electrode technique, which produces these peaks [51]. LED@CS NPs, which contain chitosan nanoparticles, lower the LED′s oxidation potential (*E*_p1_ = 0.877 V) and current (*I*_p1_ = 1.088 A). This activity is explained by chitosan’s conductive and biocompatible qualities, which stabilize the LED molecules and promote electron delivery. With a larger reduction peak current (*I*_p2_ = 1.948 A) and a lower oxidation potential (*E*_p1_ = 0.819 V), LED-PLA@CS NPs show an ill-defined peak shape. This implies that the polylactic acid coating has increased the electron transfer efficiency, which could boost the surface activity and dispersion of the nanoparticles [52].

**Figure 4:**
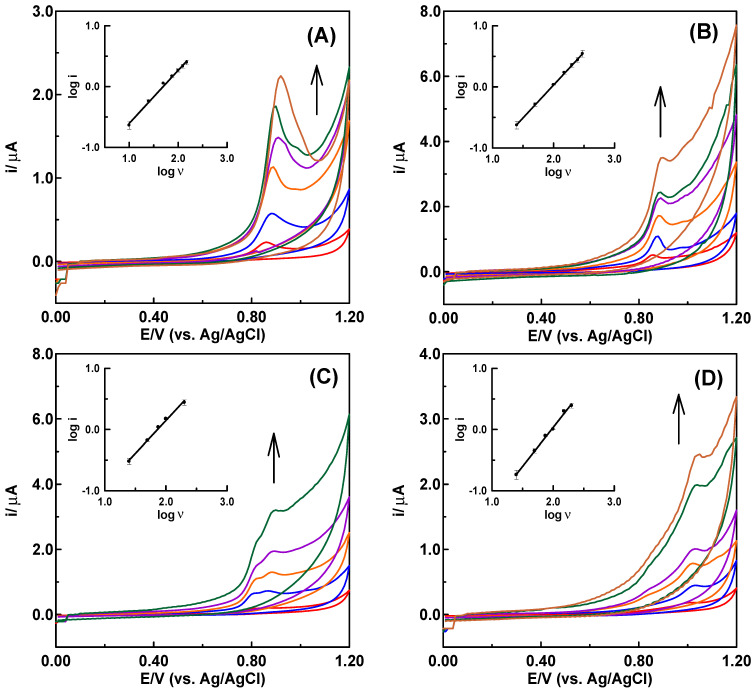
Cyclic voltammograms of 100 µM of (**A**) LED, (**B**) LED@CS NPs, (**C**) LED-PLA@CS NPs and (**D**) 30 µM of DNA in acetate buffer solution at pH 4.8 at the CPE with increasing scan rates (25 ≤ ν ≤ 200 mV/s). Insets are the log *i*_p_-log ν dependences. Arrows show the hyperchromic effects.

The CV records at different scan rates were used to analyze the features of the electrode processes involved in the electrochemical oxidation of LED, LED@CS NPs, and LED-PLA@CS NPs at the CPE surface (Figure 4). The irreversible character of the electrode process was further confirmed by Figure 4, which demonstrated that when the scan rate (v) increased, the peak potentials (*E*_p_) were shifted toward more positive values [53]. Equations (1)–(3) are given below, and graphs showing the logarithm of the peak current against the logarithm of scan rate yielded linear relations (Figure 4, insets).

LED log *i*_p_ (µA) = 0.873 log ν (mV/s) − 1.478 (r^2^ = 0.994) (1)

LED@CS NPs log *i*_p_ (µA) = 1.075 log ν (mV/s) − 2.120 (r^2^ = 0.999) (2)

LED-PLA@CS NPs log *i*_p_ (µA) = 1.078 log ν (mV/s) − 2.007 (r^2^ = 0.995) (3)

However, for the best diffusion or adsorption responses, slope values of 0.50 or 1.0 are expected [54]. The slope value of 0.873 shows that adsorption, with a contribution from diffusion, governs the electrode reaction process of LED at the CPE. The slope values of 1.075 for LED@CS and 1.078 for LED-PLA@CS NPs demonstrate that drug modification enhanced the adsorption behavior of the LED drug at the surface of CPE. Using a scan rate of 100 mV/s, the voltammogram of 30 µM DNA shows an irreversible oxidation signal at 1.032 V (Figure 4D), which is suggestive of adenine oxidation within the DNA molecule [55]. The absence of cathodic peaks in the reverse scan confirmed the irreversibility of the electrode procedure. Six electrons and six protons must be transferred in order for adenine to undergo oxidation [56]. By graphing log *i*_p_ versus log v (25 ≤ v ≤ 200 mV/s) (Figure 4D, inset), the robust interfacial characteristics of the DNA molecule at the carbon paste electrode surface were assessed. The linear relationship that resulted was explained by the following equation: log *i*_p_ (µA) = 1.274 log ν (mV/s) − 2.514 (r^2^ = 0.996).

### 2.2. DNA-Binding Studies

#### 2.2.1. Electrochemical Titration

**Figure 5:**
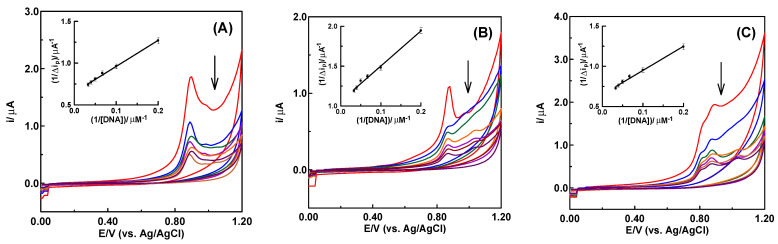
Cyclic voltammograms of increasing additions of DNA (5, 10, 15, 20, 25 and 30 µM) to 100 µM of (**A**) LED, (**B**) LED@CS and (**C**) LED−PLA@CS in acetate buffer at pH 4.8, applying a scan rate of 100 mV/s at a carbon paste electrode. Arrows show the hypochromic effects.

It was concluded from the electrochemical investigation mentioned above that these formulations may exhibit notable alterations in their redox properties in the presence of DNA due to the redox active sites. This is due to the fact that research on drug–DNA binding is crucial to comprehending the reaction mechanism and creating novel, tailored medications. Physiological pH levels of 7.4 or 4.8 are commonly used for drug–DNA interaction studies [56]. The binding of LED, LED@CS NPs, and LED-PLA@CS NPs with ct-DNA in a pH 4.8 acetate buffer solution was investigated using cyclic voltammetry. The cyclic voltammograms of a fixed concentration of 100 µM of each of the LED, LED@CS, and LED-PLA@CS NPs are displayed in Figure 5, both with and without (red curve) increasing DNA concentrations (5, 10, 15, 20, 25, and 30 µM). DNA significantly lowers the peak currents (*i*_p_) and adjusts the peak potentials (E_p_) to less positive (more negative) values when added to a solution containing LED, LED@CS, and LED-PLA@CS NPs (Figure 5). Due to interactions between the DNA and the electroactive centers (-NH of the amide groups) of LED, LED@CS NPs, and LED-PLA@CS NPs molecules, electrochemically inert DNA-LED, DNA-LED@CS NPs, and DNA-LED-PLA@CS NPs complexes grew in the solution as the DNA concentration rose. Consequently, a drop in the equilibrium concentration of unbounded medicines can be attributed to the decrease in *i*_p_. According to the literature, small molecules can bind to DNA in three different ways: (1) through non-selective electrostatic interactions with the sugar–phosphate backbone; (2) through hydrophobic forces, hydrogen bonds, Van der Waals, or electrostatic interactions within the major or minor grooves, which may distort the Watson–Crick geometry of the DNA; and (3) through intercalative binding, in which planar heterocyclic groups insert themselves between the DNA base pairs through π–π stacking interactions [57].

The change in the formal potential of LED, LED@CS NPs, and LED-PLA@CS NPs in the presence of DNA can be used to clarify the mechanism of the interactions between DNA and LED, LED@CS NPs, and LED-PLA@CS NPs. When a medication interacts electrostatically with DNA [57,58] or grooves into it [59], the formal potential typically shows a negative shift (cathodic shift) and a positive shift (anodic shift) when the drug intercalates with DNA. That LED, LED@CS NPs, and LED-PLA@CS NPs are related to DNA via both electrostatic and groove binding modes, or that the electrostatic contribution mostly favors the groove binding of pharmaceuticals with DNA, was confirmed by the peak potential’s negative shift (Figure 5). The following formula was used to calculate the binding constants (*K*) of the DNA-LED, DNA-LED@CS NPs, and DNA-LED-PLA@CS NPs complexes based on cyclic voltammetric data [59,60,61].

(4) $$\frac{1}{\Delta i_{p}}=\frac{1}{\Delta i_{p}^{max}}+\left(\frac{1}{K\;\Delta i_{p}^{max}}\right)\;\frac{1}{\left[DNA\right]}$$

where $\Delta i_{p}={(i}_{p}^{0}-i_{p})$; $i_{p}^{0}$ is the drug’s peak current when DNA is not present, $i_{p}$ is the DNA–drug complex’s peak current and $i_{p}^{max}$ is the maximum difference between the peak currents. On plotting with 1/[DNA] for DNA-LED, DNA-LED@CS NPs, and DNA-LED-PLA@CS NPs complexes, straight lines were obtained (Figure 5, insets) with the regression Equations (5)–(7). Straight lines were produced (Figure 5, insets) when 1/Δ*i*_p_ was plotted against 1/[DNA] for the DNA-LED, DNA-LED@CS NPs, and DNA-LED-PLA@CS NPs complexes using the regression Equations (6)–(7).

DNA-LED 1/Δ*i*_p_ (µA^−1^) = 3.122 {1/[DNA]} (mM^−1^) + 0.649 (r^2^ = 0.996) (5)

DNA-LED@CS NPs 1/Δ*i*_p_ (µA^−1^) = 4.409 {1/[DNA]} (mM^−1^) + 1.061 (r^2^ = 0.994) (6)

DNA-LED-PLA@CS NPs 1/Δ*i*_p_ (µA^−1^) = 3.005 {1/[DNA]} (mM^−1^) + 0.648 (r^2^ = 0.993) (7)

The binding constant values (*K*) of the DNA-LED, DNA-LED@CS NPs, and DNA-LED-PLA@CS NPs complexes were determined to be 2.1 × 10^5^ M^−1^, 2.4 × 10^5^ M^−1^ and 2.2 × 10^5^ M^−1^, respectively, based on the intercept and slope values of these equations. The strongest interaction with DNA was indicated by the highest binding constant of LED@CS NPs among the studied systems, which was followed by LED-PLA@CS NPs and free LED. The improved binding seen for LED@CS NPs in comparison to free LED indicates that encapsulation within CS NPs enhances DNA interaction, most likely as a result of favorable electrostatic interactions between the negatively charged DNA backbone and the positively charged chitosan and increased local concentration. The inclusion of the polylactic acid layer, which may partially prevent LED from being accessible to the DNA or change the surface charge, may be the cause of the somewhat lower binding constant for LED-PLA@CS NPs as compared to LED@CS NPs. These binding constant values are greater than those found for several voltammetrically investigated small molecule–DNA interactions, which typically have values between 10^3^ and 10^4^ M^−1^. According to earlier research on related systems, this suggests a robust and potentially multi-modal interaction that may involve both electrostatic and intercalative binding modes [62,63,64]. These findings demonstrate the important role that surface modification and nanoparticle encapsulation play in drug–DNA interaction, which may have consequences for the development of more potent drug delivery systems that target genetic material.

#### 2.2.2. Analysis of Thermal Denaturation

Thermal denaturation studies are integral to understanding DNA–protein interactions, as they reveal how DNA transitions from a double-stranded structure to single strands upon heating. This process, known as melting, significantly influences the binding dynamics of proteins to DNA [65]. The denaturation temperatures (*T*_m_) of free DNA, DNA-LED, DNA-LED@CS NPs, and DNA-LED-PLA@CS NPs differ noticeably, according to the results shown in Table 2. The DNA melting temperature is marginally lowered by complexation with LED and encapsulation in CS NPs as compared to free DNA. Nevertheless, additional PLA encapsulation considerably raises the melting point, indicating improved thermal stability. Changes in the DNA hydration and the immediate environment may be the cause of the modest drop in *T*_m_ that occurs with LED binding and CS NP encapsulated with LED [65]. The observed decrease in *T*_m_ for the DNA-LED and DNA-LED@CS NPs complexes is consistent with previous research that showed that the partial disruption of base stacking or changes in the hydration shell can result in decreased thermal stability in DNA–protein or DNA–drug complexes [65]. The substantial rise in *T*_m_ for the DNA-LED-PLA@CS NPs complex (75.2 °C), on the other hand, indicates that the extra PLA coating acts as a protective barrier, potentially decreasing water accessibility and boosting hydrophobic interactions. These findings demonstrate that nanoparticle encapsulation can preserve or improve DNA stability, which is essential for gene therapy, medication delivery, and biosensing [66,67].

#### 2.2.3. UV−Vis Absorption Spectral

**Figure 6:**
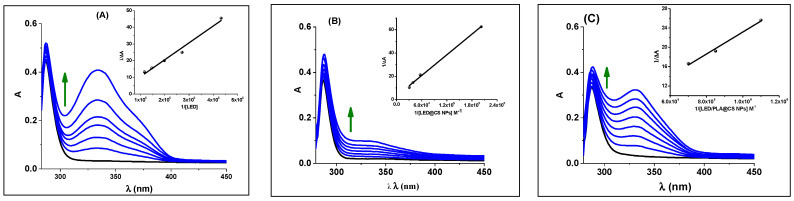
UV–vis absorption spectral changes for DNA in the absence (black lines) and presence (blue lines) of (**A**) LED, (**B**) LED@CS NPs and (**C**) LED-PLA@CS NPs at room temperature. Arrows show the absorbance changes upon increasing the LED, LED@CS NPs, and LED−PLA@CS NPs. The double reciprocal plot to determine the binding constant is shown in the inset. Arrows show the hyperchromic effects.

DNA serves as a significant target in cancer therapy, as numerous chemotherapy agents are known to interact with it [34,35]. To design and improve drugs that have better selectivity and efficacy in affecting critical biological processes, research into understanding the interactions between small molecules and DNA is essential [68]. The absorption method is a more straightforward and effective way to obtain additional clues about the manner of interaction and binding strength [69]. The UV–vis absorption spectra of ct-DNA at 250–340 in the absence and presence of increasing concentrations of LED and its encapsulated formulations (LED@CS NPs and LED-PLA@CS NPs) are reported in Figure 6. As Figure 6 illustrates, LED and LED-PLA@CS NPs exhibit considerable hyperchromism in their absorption spectra when compared to LED@CS NPs, suggesting a higher susceptibility to DNA binding. The particular spectral characteristics of DNA due to its double helical structure are hyperchromicity and hypochromicity. The intercalative binding mode is responsible for the hypochromic effect, whereas the electrostatic binding mode or partial uncoiling of the DNA helix structure, which exposes more DNA bases and indicates a strong binding of the chemicals to ct-DNA, is responsible for the hyperchromic effect [70]. Furthermore, the hyperchromic effect observed after the complex–DNA interaction indicates the alterations in the secondary structure of DNA conformation.

#### 2.2.4. Analysis of *K_b_* and Δ*G*

The intrinsic binding constants (*K*_b_) and Gibbs free energy changes (Δ*G*) are crucial parameters for comprehending the strength and thermodynamics of these interactions. The *K*_b_ was calculated by tracking the changes in the absorbance of the π–π* bands with an increasing concentration of the compound under study (Equation (8) [71]) in order to compare quantitatively the effect of the binding strength of complexes. In comparison to the nanoparticle formulations, free LED has the lowest *K*_b_ value (0.31 × 10^4^ M^−1^), suggesting a poorer interaction with ct-DNA. Encapsulation in CS NPs increases the binding affinity (0.97 × 10^4^ M^−1^), most likely as a result of the nanoparticles’ enhanced stability, solubility, or more advantageous interaction interface. The maximum *K*_b_ (1.78 × 10^4^ M^−1^) is obtained with further modification with polylactic acid (LED-PLA@CS NPs), indicating that the PLA coating increases the interaction potential, perhaps as a result of increased hydrophobic or electrostatic contacts.

(8) $$\frac{1}{∆A}=\frac{1}{{(\varepsilon }_{b}-\varepsilon_{f})L_{T}}+\frac{1}{{(\varepsilon }_{b}-\varepsilon_{f})L_{T}K_{a}[M]}$$

The spontaneous interaction between ct-DNA and LED or its formulations is confirmed by the approximate values of the Gibbs energy change (Δ*G* = −RT ln*K*_b_) for LED, LED@CS, and LED-PLA@CS NPs, which are −19.9, −22.7, and −24.3 kJ mol^−1^, respectively. This pattern implies that encapsulation improves the thermodynamics of the interaction, in addition to strengthening the binding. Increased van der Waals forces, hydrogen bonds, or other stabilizing interactions made possible by the nanoparticle matrix may be the cause of the more negative values for the nanoparticle formulations. According to these findings, LED-PLA@CS NPs perform better in terms of both the binding affinity and the thermodynamic favorability, indicating that nanoparticle encapsulation greatly improves the DNA-binding capabilities of LED. These results highlight how crucial the nanoparticle design is to maximizing drug–DNA interactions for therapeutic uses.

#### 2.2.5. Stopped-Flow Kinetics Investigation

**Figure 7:**
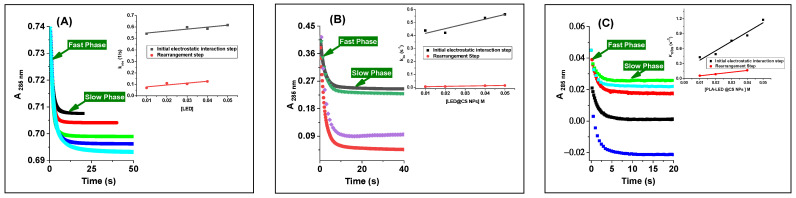
Kinetic trace at 285 nm containing two reaction steps during the reaction of DNA with (**A**) LED, (**B**) LED@CS NPs and (**C**) LED−PLA@CS NPs. Inset: plots of k_obs_ versus different concentration of formulation for the first and second reaction steps with DNA at 296 K.

The binding of drugs to duplex DNA is crucial to their antitumor activity, yet it is not the sole determinant, as many inactive compounds also show significant DNA binding. Factors such as the affinity and kinetic stability of drug–DNA complexes play a vital role in biological efficacy. Research indicates that drugs with slower dissociation rates from DNA correlate with enhanced cytotoxic effects [39,40,41,55,67,68,69,70,71,72,73,74,75]. Consequently, we employed the stopped-flow technique to assess the kinetic parameters of the binding process, which is perfect for studying rapid reaction kinetics on a millisecond timescale, to propose a reaction mechanism. The binding kinetics of LED, LED@CS NPs, and LED-PLA@CS NPs to ct-DNA can be investigated at 285 nm, as represented by the UV–vis spectra shown in Figure 6. The kinetic curves were acquired over 50 s, 40 s and 20 s for LED, LED@CS NPs, and LED-PLA@CS NPs, respectively, covering the entire process (Figure 7). All the kinetic curves were fitted to a bi-exponential function, indicating the involvement of at least a two-step process in the binding of LED, LED@CS NPs, and LED-PLA@CS NPs to ct-DNA. Additional steps may be identified during the binding process, but they do not influence the absorbance. The reaction of LED, LED@CS NPs, and LED-PLA@CS NPs with ct-DNA shows a fast decrease in absorbance, followed by a slow decrease. To investigate the concentration dependency of the observed reaction rates, 10^−4^ M solutions of ct-DNA were reacted with varying concentrations of LED, LED@CS NPs, and LED-PLA@CS NPs, while keeping the pseudo-first-order condition. The second-order association constant (*k*_on_), first-order dissociation constant (*k*_off_), equilibrium association constants (*K*_a_ [M^−1^] = (*k*_on_/*k*_off_)), equilibrium dissociation constants (*K*_d_ [M] = *k*_off_/*k*_on_), and Gibbs free energy (Δ*G* [kJ mol^−1^]) for these interactions were determined.

#### 2.2.6. Analysis of the Fast Phase

The first step, generation of binary intermediates through electrostatic interactions, shows a notable improvement in the binding kinetics. From free LED (2.5 ± 0.3 M^−1^ s^−1^) to LED@CS NPs (5.72 ± 0.60 M^−1^ s^−1^), the association rate constant (*k*_1_) gradually rises until reaching its maximum with LED-PLA@CS NPs (18.2 ± 2.2 M^−1^ s^−1^), which is a ~7.3-fold increase over free LED (Table 3). This significant increase in the association rate implies that encapsulation of nanoparticles, especially with the PLA@CS composite method, promotes initial binding events with ct-DNA and more effective molecular recognition. At the same time, the dissociation rate constant (*k*₋_1_) drops from 0.56 ± 0.01 s^−1^ for free LED to 0.22 ± 0.02 s^−1^ for LED@CS NPs and 0.15 ± 0.07 s^−1^ for LED-PLA@CS NPs. When LED is encapsulated, the initial DNA–drug combination is more stable, as evidenced by the progressive decrease in *k*₋_1_. The most stable initial binding is shown by the LED-PLA@CS NPs. A ~27-fold increase in the binding affinity for the PLA@CS composite nanoparticles is represented by these kinetic parameters, which result in a substantial rise in the equilibrium association constant (*K*_a1_) from 4.48 M^−1^ for free LED to 26.0 M^−1^ for LED@CS NPs and 120 M^−1^ for LED-PLA@CS NPs. This trend is further supported by the equivalent drop in the *K*_d1_ values, which went from 22.0 × 10^−2^ M to 0.83 × 10^−2^ M. The increase in the negative Gibbs free energy values (Δ*G*_1_), from −3.72 to −11.85 kJ mol^−1^, shows that nanoparticle encapsulation makes the first binding step much more thermodynamically advantageous.

#### 2.2.7. Analysis of the Slow Phase

More complex kinetic behavior is displayed in the second step, which involving synchronous internal rearrangement. Interestingly, the *k*_2_ value of LED@CS NPs is lower (0.24 ± 0.02 M^−1^ s^−1^) than that of free LED (1.63 ± 0.58 M^−1^ s^−1^); however, the maximum value is displayed by LED-PLA@CS NPs (3.58 ± 0.3 M^−1^ s^−1^) (Table 4). This non-linear trend implies that the conformational flexibility needed for the rearrangement step may be limited by CS nanoparticles alone, but PLA addition seems to promote this process, perhaps by offering the best molecular orientation or microenvironment for the rearrangement. In comparison to free LED (6.0 ± 1.0 × 10^−2^ s^−1^), the dissociation rate constants for the second step (*k*₋_2_) drop by around 20 times for LED@CS NPs (0.3 ± 0.1 × 10^−2^ s^−1^), with LED-PLA@CS NPs exhibiting an intermediate value (2.0 ± 0.7 × 10^−2^ s^−1^). Both nanoparticle compositions have enhanced *K*_a2_ values as a result, with LED-PLA@CS NPs obtaining the highest value (179.0 M^−1^), which is roughly 6.6 times higher than free LED (27.2 M^−1^). Similar to the first step, the second step’s Gibbs free energy values (Δ*G*_2_) show the increased thermodynamic favorability of the rearrangement process in the nanoparticle systems, going from free LED (−8.14 kJ mol^−1^) to LED@CS NPs (−10.81 kJ mol^−1^) and LED-PLA@CS NPs (−12.80 kJ mol^−1^).

#### 2.2.8. Analysis of the Overall Reaction

The overall association constant, *K*_a_, can be determined by calculating the overall equilibrium dissociation constant, *K*_d_, from the individual equilibrium dissociation constants, *K*_d1_ and *K*_d2_, using Equation (9). The overall binding free energy change G_bind_ can also be obtained using Equation (10).

(9) $$K_{d}=\frac{K_{d1}K_{d2}}{1+K_{d2}}$$

(10) $$∆G_{bind}=RT\cdot ln{(K}_{d})$$

**Scheme 2:**
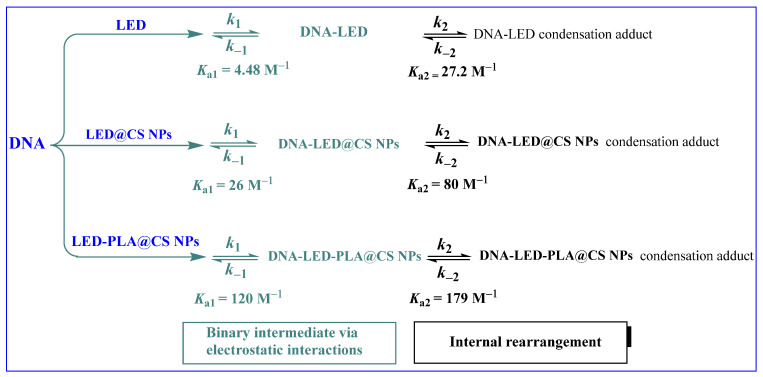
Interaction mechanism of LED, DNA−LED@CS NPs, and DNA−LED−PLA@CS NPs with DNA.

Overall, the *K*_d_ values drop by around 173 times from free LED (7.8 × 10^−3^ M) to LED-PLA@CS NPs (0.045 × 10^−3^ M), whereas the *K*_a_ values rise by about 172 times from 128.4 M^−1^ to 22026 M^−1^. Compared to −12.02 kJ mol^−1^ for free LED, the overall Δ*G* values for LED-PLA@CS NPs become noticeably more negative, reaching −24.79 kJ mol^−1^ (Table 5). According to our results, the thermodynamic and kinetic favorability of LED binding to ct-DNA are significantly improved by nanoparticle encapsulation, especially when using the PLA@CS composite nanoparticles method. According to the stopped-flow study, this improvement results in much better binding affinity by increasing the association rates and decreasing the dissociation rates in both binding phases. Following an initial electrostatic interaction between the positively charged nanoparticle surface and the negatively charged DNA phosphate backbone, the two-step binding mechanism described here most likely entails a rearrangement that may involve partial intercalation, groove binding, or conformational changes in both the drug–nanoparticle complex and the DNA (Scheme 2). Perhaps as a result of a synergistic effect, the PLA@CS nanoparticles seem to maximize both processes: the PLA component allows for optimal rearrangement and stabilization of the final complex, while the CS component facilitates the initial electrostatic interaction.

#### 2.2.9. Analysis of Drug–DNA Kinetic Stability

**Figure 8:**
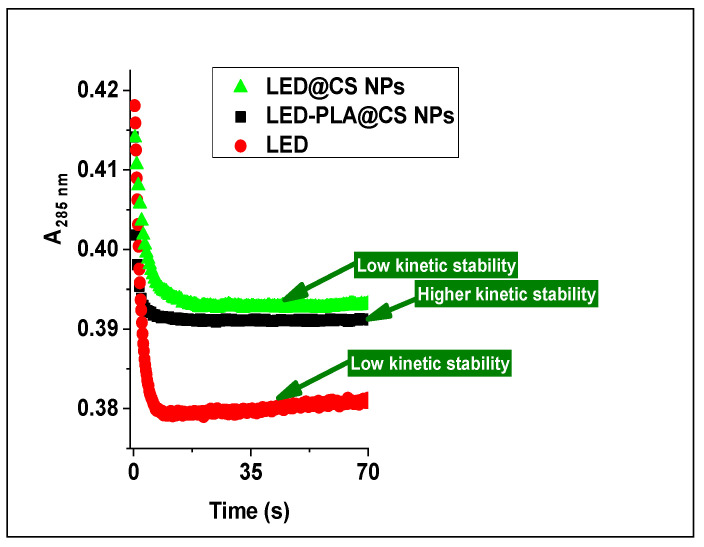
Kinetic trace showing the stability of DNA-LED, DNA-LED@CS NPs and DNA-LED-PLA@CS NPs complexes over longer.

Interestingly, the results also suggest that nanoparticle encapsulation improves the kinetic stability of the drug–DNA complex, particularly when employing the PLA@CS composite nanoparticles system, as shown in Figure 8. The DNA-LED-PLA@CS NPs and DNA-LED@CS NPs complexes showed higher stability over a longer time span than free LED. These mechanistic results from the stopped-flow kinetic analysis provide crucial information for the rational design of nanoparticle drug delivery systems targeting nucleic acids, which may find applications in gene therapy, antiviral treatments, and anti-cancer medication development.

#### 2.2.10. Evaluation of Nanoparticle–DNA Binding Affinity

The kinetic stability of DNA interactions with LED@CS NPs and LED-PLA@CS NPs was evaluated through cyclic voltammetry (CV), UV–vis spectroscopy, and stopped-flow kinetics to elucidate the enhanced binding affinity observed in the nanosystems compared to free LED. Cyclic voltammetry revealed that LED-PLA@CS NPs exhibited a lower oxidation potential and higher peak current (E_p1_ = 0.819 V, I_p2_ = 1.948 A) compared to LED@CS NPs (E_p1_ = 0.877 V, I_p1_ = 1.088 A), suggesting improved electron transfer efficiency due to the PLA coating. This enhanced electrochemical behavior correlates with the higher binding constant of LED-PLA@CS NPs (*K*_a_ = 22026 M^−1^) compared to LED@CS NPs (*K*_a_ = 2131 M^−1^), indicating stronger DNA interaction. However, the reduced zeta potential of LED-PLA@CS NPs (+33.27 mV) compared to LED@CS NPs (+43.51 mV) suggests that the surface charge is not the primary driver of this enhanced binding. Instead, the PLA layer likely promotes hydrophobic interactions and structural stabilization, facilitating stronger intercalative binding with ct-DNA, as confirmed by UV–vis spectroscopy and stopped-flow kinetics. These findings indicate that the PLA modification enhances the kinetic stability of nanoparticle–DNA complexes, likely through a combination of hydrophobic and intercalative interactions, supporting the potential of LED-PLA@CS NPs for targeted therapeutic applications.

### 2.3. Loading Efficiency

Because of their improved bioavailability, regulated drug release, and effective payload capacity, CS NPs are being utilized more and more [76]. Polylactic acid (PLA) and its copolymers are biocompatible, bioabsorbable, biodegradable via hydrolysis and enzymes, and offer diverse mechanical properties for various applications with low immunogenicity. FDA-approved PLA formulations enhance clinical translatability [77]. Combining PLA with chitosan (CS) improves the hydrophilicity, reduces the initial burst release, enhances the protein adsorption, and addresses the slow degradation [78]. Polymeric nanocarriers provide consistent drug release, a longer systemic half-life, and a higher drug payload compared to other types [79]. According to the UV–vis investigation, the LED-loading efficiency rose as the LED concentration rose. For CS NPs and PLA@CS NPs, the loading content of LED was found to be approximately 13.48% and 71.43% (for 10 ppm), respectively, while the loading level of LED peaked at 44.06% and 89.3% (for 50 ppm). The higher drug-loading efficiency of LED-PLA@CS NPs compared to LED@CS NPs can be attributed to the hydrophobic nature of PLA, which enhances the encapsulation of the poorly water-soluble ledipasvir through hydrophobic interactions. The *w*/*o*/*w* emulsion technique used for LED-PLA@CS NPs allows for better entrapment of the drug within the PLA core, while the chitosan shell provides additional stability and prevents premature drug leakage. This dual-polymer system creates a more robust matrix, increasing the drug payload capacity compared to the ionotropic gelation method used for LED@CS NPs alone. PLA@CS NPs is a promising vehicle and efficient method for our drug delivery because of its higher drug-loading efficiency.

### 2.4. In Vitro Stimuli-Responsive Release

Nanoparticles (NPs) improve the safety and efficacy by extending the circulation, assisting with membrane transport, and increasing the medication stability and solubility. Long-term drug concentration maintenance in the blood or target tissue is achieved using controlled-release devices [80]. Normal physiological conditions and the intracellular environment of a tumor were simulated by pH values of 7.4 and 5.4 (with 10 mM GSH), respectively. Because chitosan protonation causes swelling, the acidic milieu of tumors—which is brought on by poor vascular architecture and metabolic byproducts—causes a faster release of the NP content; increased acidity raises the concentration of H^+^, which speeds up the release of LED [81]. Tumor cells exhibit a glutathione (GSH) concentration 4–5 times greater than that of normal cells, with the levels ranging from 2 to 10 mM in the cytoplasm [82].

**Figure 9:**
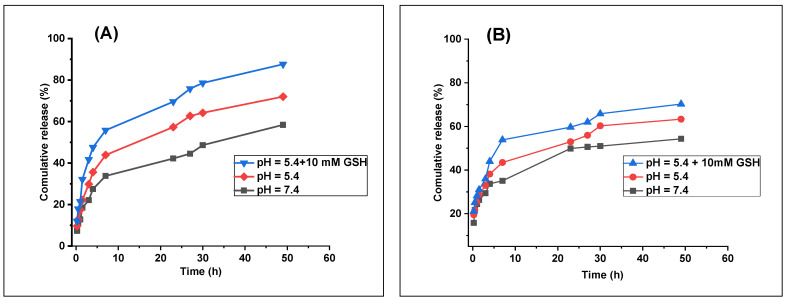
In vitro drug release profiles of (**A**) LED@CS NPs and (**B**) LED-PLA@CS NPs in PBS at pH 7.4, pH 5.4, and pH 5.4 with 10 mM GSH at 37 °C (*n* = 1).

The LED release patterns from LED@CS NPs and LED-PLA@CS NPs under physiological pH (7.4), acidic pH (5.4), and acidic pH with 10 mM GSH are displayed in Figure 9. Free LED was not included as a reference in the release studies because its poor aqueous solubility and rapid dissolution in physiological conditions make it unsuitable for direct comparison with the controlled-release profiles of the nanoparticle formulations [29]. The focus was on evaluating the stimuli-responsive release of encapsulated LED under tumor-mimetic conditions. The behavior that responds to stimuli is highlighted by the release profiles. The LED release from LED@CS NPs and LED-PLA@CS NPs was only 33% and 35% within 7 h at pH 7.4, respectively. This suggests delayed release and stability, which are essential for reducing premature drug release. The release rate for LED@CS NPs and LED-PLA@CS NPs dramatically rose to 43% and 43.5%, respectively, at an acidic pH (5.4), which mimics the tumor environment. This suggests that chitosan protonation is how the NPs respond to acidic environments. For all the formulations, the addition of 10 mM GSH at pH 5.4 further increased the LED release to roughly 55% and 53%, respectively. Nearly 88% of the LED was released from LED@CS NPs and 73% from PLA-LED@CS NPs after 54 h at pH 5.4 with GSH. This was explained by the reduction-sensitive GSH-responsive connections that were broken by the increased GSH levels in tumor cells [83]. By taking advantage of the tumor acidity and reductive conditions, the enhanced release demonstrates the possibility of site-specific medication delivery. These results support stimulus-triggered LED release in cancer cells by demonstrating the sensitivity of drug-loaded CS NPs to the pH and GSH [84,85]. It is possible that polylactic acid (PLA) reduces the drug release efficiency because of variations in the nanoparticle composition or structural characteristics, as seen by the consistently decreased cumulative release percentages for LED-PLA@CS NPs. In order to minimize the adverse effects and increase the efficacy, the dual responsiveness effectively releases their payload in tumors while maintaining stability in normal conditions.

### 2.5. In-Vitro Drug Release Mechanism

**Figure 10:**
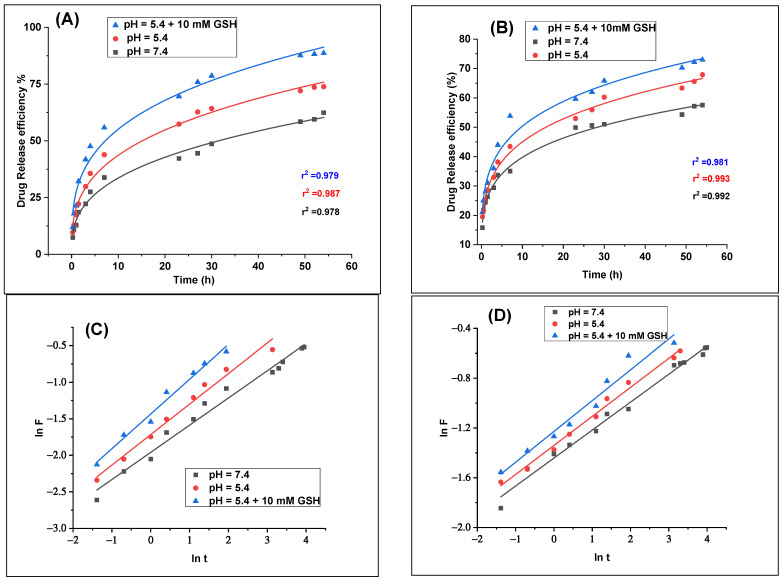
LED release fitting curves for (**A**) LED@CS NPs and (**B**) LED−PLA@CS NPs according to the Korsmeyer–Peppas model. Representation of the first 60% of drug fractionally released from (**C**) LED@CS NPs and (**D**) LED−PLA@CS NPs according to the Korsmeyer–Peppas equation (*n* = 1).

Because it enables us to estimate the release kinetics before creating release systems, mathematical modeling is quite advantageous. Key physical characteristics, such the drug diffusion coefficient and experimental release data, are typically measured in order to develop models. Understanding the drug release kinetics is essential for mathematical modeling, as it impacts all the formulations and is essential for enhancing the solution profiles [86]. We employed models such as the first-order, second-order, Higuchi, and Korsmeyer-Peppas models (Figure 10A,B and Supporting Information) to determine the optimal drug release mechanism. The Korsmeyer–Peppas model was identified as the best fit for modeling the drug release kinetics, based on the highest correlation coefficient (r^2^) (Table 6). At pH values of 7.4, 5.4, and 5.4, including 10 mM GSH, the r^2^ values for LED@CS NPs were 0.979, 0.987, and 0.978, and for LED-PLA@CS NPs, they were 0.992, 0.993, and 0.981 (Figure 9A,B and Table 6).

### 2.6. Analysis of K_kp_ and n

The Korsmeyer–Peppas model was used to examine the release kinetics of LED from CS NPs at 37 °C, both with and without PLA. There was a significant correlation between the experimental data and Equation (17), as indicated by the relatively high correlation coefficient (r^2^) for each set of data, which ranged from 0.979 to 0.993. In order to simulate various physiological conditions, we also plotted the ln of the cumulative percentage of drug released against the ln of time. The kinetic constant (*K*_kp_) and release exponent (*n*) were calculated from Equation (11) at pH 7.4, pH 5.4, and pH 5.4 with 10 mM glutathione (GSH) (Figure 10C,D) [87,88].

(11) $$F=\frac{M_{t}}{M_{\infty }}=K_{kp}t^{n}\to lnF=lnK_{kp}-lnt$$

where n is a dimensionless exponent that aids in explaining how the drug is released, *K_kp_* is a constant that influences the release rate, *t* is the time, and *M_t_/M_∞_* is the fraction of drug released. While the constant K_kp_ provides information on the drug formulation and the characteristics of the nanocarriers, the exponent “*n*” is crucial since it tells us about the release mechanism. In LED@CS NPs and LED-PLA@CS NPs, the parameter *K* denotes constant drug transport, which is precisely connected to the drug release kinetics. In actuality, a higher *K* indicated quicker drug release. Conversely, a lower *K* value indicates ineffective drug release from nanocarriers due to a poor transport kinetic.

With 10 mM GSH, the release exponent (*n*) for LED@CS NPs varied from 0.37 at pH 7.4 to 0.48 at pH 5.4. The *K*_kp_ values similarly rose with a decreasing pH and the presence of GSH, from 0.14 at pH 7.4 to 0.24 at pH 5.4 with 10 mM GSH. This implies that the protonation of chitosan and disruption of the nanoparticle matrix may be the reasons why the release of LED from CS NPs is more advantageous in acidic environments and when a reducing agent such as GSH is present. The LED-PLA@CS NPs, however, displayed a distinct pattern. With 10 mM GSH, the release exponent (*n*) ranged from 0.22 at pH 7.4 to 0.24 at pH 5.4, which was lower than that of LED@CS NPs. The *K*_kp_ values, which ranged from 0.24 at pH 7.4 to 0.29 at pH 5.4 with 10 mM GSH, were generally greater than those of LED@CS NPs. Perhaps by forming a more stable matrix that slows down the drug release, the inclusion of PLA seems to have changed the release mechanism. The four methods of drug release are as follows: (i) Fickian diffusion (*n* < 0.5), (ii) non-Fickian (0.5 < *n* < 1), (iii) zero order (*n* = 1), or non-Fickian super release (*n* > 1), according to P. Costa et al. [89]. In our case, the *n* values for the release in all the pH mediums are less than 0.5, as determined by Equation (11), and are shown in Table 7. This is consistent with a Fickian diffusion process, in which diffusion through the polymer matrix mostly regulates the drug release. Given that the solvent mobility is incredibly low compared to the relaxation rate, this pattern indicates that the release mechanism is entirely controlled by diffusion [90]. The outcomes of the discharge will guarantee that the medications have the greatest potential impact on cancerous cells.

### 2.7. Antibacterial Activity

**Figure 11:**
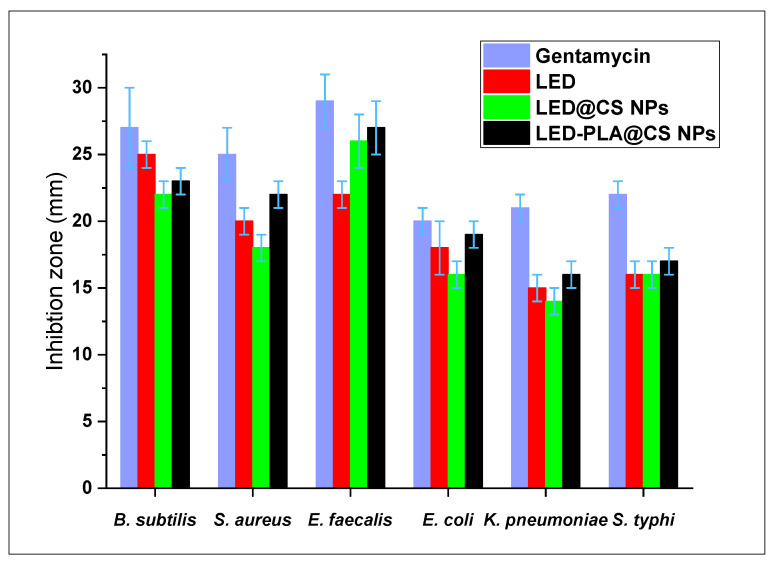
Antibacterial activity of LED, LED@CS NPs, and LED–PLA@CS NPs against Gram-positive and Gram-negative bacteria, with gentamycin as a reference (*n* = 3, mean ± SD). Error bars represent standard deviation.

Gram-positive (*Bacillus subtilis*, *Staph. Aureus*, *Enterococcus faecalis*) and Gram-negative (*Escherichia coli*, *K. pneumonia*, *Salmonella typhi*) bacteria were used to assess the antibacterial activity of free LED and its encapsulated formulations in vitro. Gentamycin was used as a reference (Figure 11). The zone of inhibition figures show how well each formulation works to stop the development of bacteria. Free LED showed modest effectiveness against Gram-positive bacteria, with zones of inhibition ranging from 20 ± 1 mm to 22 ± 1 mm. When LED was encapsulated in chitosan nanoparticles (LED@CS NPs), the activity against *S. aureus* was marginally decreased, but the effect against *B. subtilis* (22 ± 1) and *E. faecalis* (26 ± 2 mm) were increased. When compared to free LED and LED@CS NPs, the PLA coating (PLA-LED@CS NPs) increased the activity against all the tested bacteria. Generally, all the LED formulations had less antibacterial action against Gram-negative bacteria than against Gram-positive bacteria. The lack of a protective outer membrane (lipopolysaccharide and protein) in G+ bacteria may account for this, as it reduces their resistance to antibacterial drugs [91]. Overall, the activity of LED-PLA@CS NPs was marginally better than that of free LED and LED@CS NPs.

### 2.8. Cytotoxic Activity

**Figure 12:**
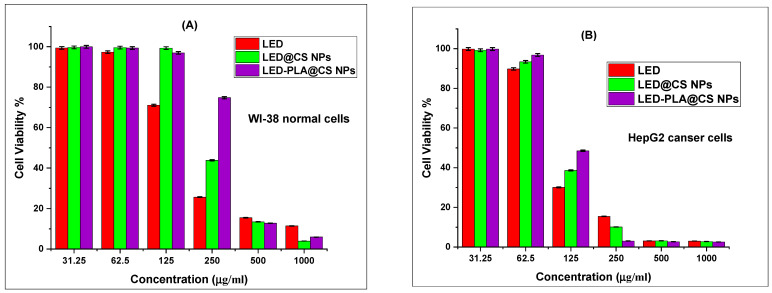
Cell viability results from the MTT assay for (**A**) WI-38 normal cells and (**B**) HepG2 cancer cells with LED, LED@CS NPs, and LED–PLA@CS NPs for 24 h (*n* = 3, mean ± SD). Error bars represent standard deviation.

As demonstrated in Table 8 and Figure 12A,B, the cell viability findings demonstrated that the cytotoxicity of LED, LED@CS NPs, and LED-PLA@CS NPs is concentration-dependent across the tested range of 31.5 μg/mL to 1000 μg/mL. The IC_50_ value of free LED for HepG2 cells was 101.76 ± 0.33 µg/mL. When LEDs were encapsulated in CS NPs (LED@CS NPs), the IC_50_ increased marginally to 110.46 ± 1.35 µg/mL. When polylactic acid (LED-PLA@CS NPs) was added, the IC_50_ value increased more noticeably to 144.76 ± 0.69 µg/mL. This implies that the encapsulation and PLA alteration may decrease LED’s instant availability, which could have an impact on its immediate cytotoxicity in HepG2 cells. On the other hand, in all the samples, the IC_50_ values in WI-38 cells were significantly greater. The IC_50_ values for LED@CS NPs and LED-PLA@CS NPs were 232.85 ± 2.63 µg/mL and 341.77 ± 3.32 µg/mL, respectively, whereas the IC_50_ value for free LED was 184.46 ± 2.67 µg/mL. For all the studied formulations, the higher IC_50_ values in WI-38 cells relative to HepG2 cells indicate that these formulations are less harmful to healthy cells. Due to the different features of the cell membranes and energy metabolism in normal and cancer cells, endocytosis—the mechanism through which nano-formulations penetrate cells—may be less efficacious in normal cells [92]. These findings imply that encasing LED in CS NPs and altering it with PLA may alter its cytotoxic properties, possibly increasing its selectivity for cancer cells and decreasing its toxicity for healthy cells.

### 2.9. Analysis of Therapeutic Index

The therapeutic index (TI) can be calculated by dividing the IC_50_ value in normal cells (WI-38) by the IC_50_ value in cancer cells (HepG2) [93], using the IC_50_ values for HepG2 and WI-38 cells as a guide. A greater margin of safety is indicated by a higher therapeutic index. A TI greater than one indicates some selectivity for cancer cells over healthy cells in all the formulations. When LED is converted into LED@CS NPs and then LED-PLA@CS NPs, the therapeutic index rises even more (Table 8). This suggests that the drug’s safety profile is enhanced by encapsulation and PLA modification, possibly by the decrease in the drug’s toxicity to healthy cells while preserving its effectiveness against cancer cells. When compared to free LED or LED@CS NPs, PLA modification results in the largest increase in the TI, suggesting that PLA helps achieve a more targeted delivery or a less off-target effect. The reticuloendothelial system (RES) may impact nanoparticle delivery by clearing particles from circulation, particularly those with sizes >200 nm, such as LED-PLA@CS NPs (393.7 nm). The chitosan coating may reduce the RES uptake due to its mucoadhesive and biocompatible properties [22], but PEGylation or other stealth modifications could further enhance the circulation time and tumor accumulation. In vivo studies are needed to quantify the RES clearance and optimize the delivery efficiency. The finding of an increased therapeutic index (TI) among the formulations—LED (1.8), LED@CS NPs (2.1), and LED-PLA@CS NPs (2.4)—is especially noteworthy. The stability constants (*K*_a_) demonstrate a strong correlation between this improvement and an increase in the DNA-binding affinity: LED (128.4 M) < LED@CS NPs (2131 M) < PLA@CS NPs LED (22026 M). The thermodynamic data provides further information on the binding mechanisms. The decreasing Gibbs free energy values (ΔG°) from LED (−12.02 kJ/mol) to LED@CS NPs (−18.98 kJ/mol) to LED-PLA@CS NPs (−24.79 kJ/mol) show increasingly more spontaneous and energetically favorable DNA interactions. This enhanced binding stability could affect the nano-formulations’ prolonged drug-releasing characteristics. The results of the study on the DNA-binding affinity are especially significant since they provide a mechanistic understanding of ledipasvir’s activity outside its well-known antiviral effects. The consistent rise in the therapeutic index across the formulations suggests that nanoencapsulation methods could offer practical means to repurpose ledipasvir outside of its current use in the treatment of HCV, possibly extending its use to cancer therapy with improved safety profiles. To clarify the reasons behind these variations and assess the safety and effectiveness of these nano-formulations in vivo, more research is planned.

### 2.10. Morphological Analysis

**Figure 13:**
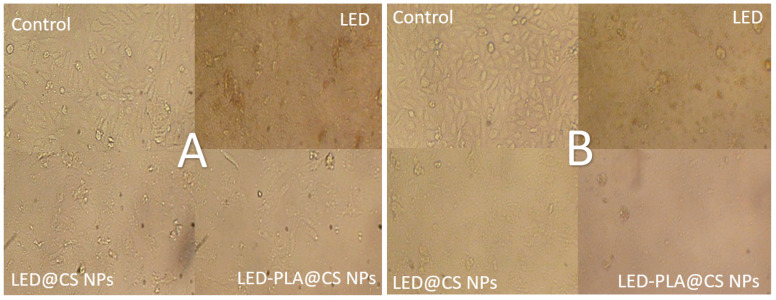
Morphological changes in (**A**) WI-38 normal cells and (**B**) HepG2 cancer cells following 24 h of treatment with LED, LED@CS NPs, and LED-PLA@CS NPs at 1000 µg/mL (*n* = 3). Scale bar: 25 µm.

The morphological alterations seen WI-38 normal cells and in HepG2 cancer cells following a 24 h treatment with different formulations of LED at a concentration of 1000 µg/mL are depicted in Figure 13A,B. All the treatment cell lines showed only slight morphological changes in WI-38 normal cells as compared to the control. This implies that at the studied dose, the treatments—including the nanoparticle formulations—did not significantly harm normal cells. On the other hand, HepG2 cancer cells responded to the therapies with noticeable morphological changes. In particular, when compared to the control and free LED, cells treated with LED@CS NPs and LED-PLA@CS NPs displayed symptoms of cellular stress, including cell shrinkage and changed shape. This suggests that the lethal effect of LED on HepG2 cancer cells is enhanced when it is encapsulated in nanoparticles, especially when coated with polylactic acid. The enhanced cellular absorption and/or prolonged release of LEDs due to nanoparticle delivery technology could be responsible for the increased effect.

## 3. Materials and Methods

### 3.1. Chemicals

Ledipasvir was obtained from Alhayat Technology for Raw Material Industries Co. Ltd., “Technovir,” Shafa Badran, Amman, Jordan. Bio Basic Inc. (Toronto, ON, Canada) provided the methyl thiazolyl tetrazolium (MTT), ascorbic acid, and gentamycin. Sigma-Aldrich Manufacturing LLC (Saint Louis, MO, USA) provided the ct-DNA, acetic acid, trimeric sodium phosphate (TPP), and sodium hydroxide. Haixin Biological Product Co., Ltd. (Qufu, China) provided the CS, deacetylation degree 80% and molecular weight 400,000, for the study. Polylactic acid (PLA, molecular weight 60,000–80,000 Da, Alfa Chemical Group, El-Marwa Tower, Hadayek El-Kobba, Egypt) was used for the nanoparticle preparation. The human lung (WI-38) and liver cancer (HepG2) cell lines used in the study were obtained from a holding company for biological products and vaccines, Dokki, Giza, Egypt. The analytical-grade chemicals used in this experiment were all used just as they were provided.

### 3.2. Preparation of Chitosan Nanoparticles

The process for developing chitosan nanoparticles was based on the ionotropic gelation of chitosan and sodium tripolyphosphate (TPP) [94,95]. Using 0.5% (*w*/*v*) chitosan and 1% *(v*/*v*) acetic acid, the mixture was stirred for two hours at room temperature until it completely dissolved. The pH was lowered to 5.0 by adding NaOH solution. TPP was dissolved at a 0.25% (*w*/*v*) concentration in water. The addition of TPP dropwise by burette to the chitosan solution (1:3 *v*/*v*) under magnetic stirring for one day until solid CS NPs emerged helped to facilitate the formation of chitosan nanoparticles. After centrifuging the colloidal solution for ten minutes, it was twice cleaned to get rid of any unreacted material before being freeze-dried.

### 3.3. Preparation of LED-Loaded Chitosan Nanoparticles

Ledipasvir solution was added to CS and TPP during the gelation phase (incorporation) in order to produce LED@Cs NPs for this study [94]. First, 0.03 g of ledipasvir was made in 10 milliliters of 1% (*v/v*) acetic acid. Then, using a burette, the drops were added to the CS solution while being stirred for two hours. Thus, using a burette, 0.25% (*w/v*) TPP solution was added dropwise to a 1:3 *v/v* chitosan solution while being magnetically stirred for a day until solid LED@CS NPs form. The product was then freeze-dried for additional characterization and research after being cleaned to remove any remaining unreacted material.

### 3.4. Preparation of LED-PLA-Loaded Chitosan Nanoparticles

The *w*/*o*/*w* emulsion approach was used to encapsulate the medication [33,96,97]. In order to formulate a *w/o* emulsion, an aqueous drug solution (0.02 gm in 2 mL) was first added to a polymer solution (200 mg of PLA dissolved in 20 mL dichloromethane). After that, the *w/o* emulsion was quickly added to 20 milliliters of a 1% acetic acid solution that contained 80 milligrams of chitosan. After 30 min of sonication to create an emulsion, the fluid was aggressively agitated. Until the organic solvent disappeared, stirring persisted. Finally, the specimen underwent lyophilization in a freeze-dryer.

### 3.5. Characterization

The characterization, loading, release efficiency, and ct-DNA denaturation investigations were performed using a UV–vis spectrophotometer (PEAK USA model C7000V) within the 190–1100 nm range. The Fourier transform infrared (FT-IR) spectra of the nanoparticles were acquired using potassium bromide pellets on a JASCO FT/IR-6800 type A spectrometer, with a resolution of 2.0 cm^−1^ and a wave number range of 400–4000 cm^−1^, to observe both existing and newly created functional groups. X-ray diffraction analyses employing Cu-Kα radiation were performed to determine the sample’s structure, applying a Shimadzu X-ray diffractometer from Kyoto, Japan. The operational parameters for the X-ray source were 40 kV and 30 mA. In reflection mode, the diffraction intensity was assessed for 2θ = 5−80° at a scanning speed of 8°/min. The polydisperse index (PDI) of the diluted solution was determined using dynamic light scattering (DLS) with a zetasizer (Malvern Instruments, 1000 HS, Malvern, UK) in a Tris buffer solution. The hydrodynamic diameter of the samples, under the assumption of a spherical shape, was determined using dynamic light scattering with noninvasive back scattering (DLS-NIBS) at a laser wavelength of 632 nm and a scattering angle of 173 degrees. Phase analysis light scattering (M3-PALS) and mixed laser Doppler electrophoresis were employed to determine the zeta potential (ζ) in a Tris buffer solution at ambient temperature. The process was repeated three times for each analysis run to ensure consistent results. The Malvern Zetasizer Nano, Model ZS 3600 (Malvern Instruments, Worcestershire, UK), was utilized to perform both measurements. The surface morphology changes were observed using a scanning electron microscope (JSMIT100 by JEOL, Tokyo, Japan) operating at an acceleration voltage of 10 kV. A computer-controlled PalmSens 4 Potentiostat/Galvanostat/Impedance Analyzer (PalmSens BV, Randhoeve 221, 3995 GA Houten, The Netherlands), utilizing PS Trace 5 software version 5.9 (PalmSens BV), was employed for the cyclic voltammetric characterization measurements. A Hanna digital pH meter, model PH211 (Hanna Instruments, Nusfalau, Romania), with a glass combination electrode, was employed for all the pH-metric measurements. This device has been standardized using buffers with known pH values.

### 3.6. Procedures for DNA Binding Studies

#### 3.6.1. Cyclic Voltammetry Measurements

A computer-operated PalmSens 4 Potentiostat/Galvanostat/Impedance Analyzer (PalmSens BV, Randhoeve 221, 3995 GA Houten, The Netherlands), utilizing PS Trace 5 software version 5.9 (PalmSens BV), was employed for the cyclic voltammetric measurements. The micro-voltametric cell (PAR Model K0262) was made up of a carbon paste electrode body (BAS model MF-2010), an Ag/AgCl/3M KCl reference electrode (PAR K0265), and a platinum wire counter electrode (PAR model K0266). A copper wire was centrally positioned within the Teflon rod to establish contact, while the carbon paste electrode consisted of a Teflon rod featuring a 3 mm diameter end chamber and a 1 mm deep cavity at one extremity for paste infusion. To create a homogeneous graphite paste, 5.0 g of graphite powder (1–2 μm, Aldrich, Milwaukee, WI, USA) and 1.8 mL of Nujol oil (Sigma, d = 0.84 g/mL) were manually combined in an agate mortar. Following the insertion of the produced graphite paste into the Teflon rod end chamber, the electrode surface was meticulously smoothed using clean paper.

#### 3.6.2. Thermal Denaturation Measurements

The UV–visible spectroscopy technique was employed to investigate the denaturation of the ct-DNA double helix. The experiments involved tracking the absorption of a 50 μM ct-DNA sample at different temperatures with and without LED, LED@CS NPs, and LED-PLA @CS NPs. By adjusting the temperature between 30 and 100 °C, the absorbance of each complex was recorded at 288 nm. The melting temperature (*T*_m_) is the halfway of the inflection or jump in the absorbance versus temperature plot [98,99].

#### 3.6.3. UV–Vis Absorption Spectra

Using UV–vis spectroscopy, the potential binding modes of LED, LED@CS NPs, and LED-PLA@CS NPs to ct-DNA were examined, and the associated DNA-binding constants (*K*_b_) were computed. A UV–vis spectrophotometer (PEAK USA model C7000V, PEAK Electronic Development Pty Ltd., Bendigo, Australia) was used to obtain the electronic spectra of the constant DNA concentration (1 × 10^−4^ M) solution (pH = 7.4) in sodium phosphate buffer (20 mM) at room temperature (25 °C) in the wavelength range of 200–500 nm. LED, LED@CS NPs, and LED-PLA@CS NPs were applied in varying quantities, and the spectra were collected to evaluate the materials’ influence on DNA conformation.

#### 3.6.4. Stopped-Flow Experiments and Kinetic Measurements

The stopped-flow device from Applied KinetAsyst SF-61DX2 (HI-Tech Scientific, Kolkata, India) was used to assess the DNA-binding kinetics. In the stopped-flow system, equal volumes of LED, LED@CS NPs, LED-PLA@CS NPs, and DNA in buffer (50 mM Tris acetate, pH 7.5) from different syringes were quickly mixed at 25 °C. The spectral variations of the reactions were initially observed over the wavelength range of 190–600 nm in order to select an appropriate wavelength at which the kinetic traces could be followed. The compounds (LED, LED@CS NPs, and LED-PLA@CS NPs) were used in at least a 10-fold excess to study the binding reactions under pseudo-first-order circumstances. Regular variations in the protein absorbance were noted both before (t = 0 s) and after injection with LED, LED@CS NPs, and LED-PLA@CS NPs. For a 1:1 mixture, the instrument’s dead time was determined to be 1 ms. In the control trials, DNA solution was combined with buffer solutions that included either no LED, LED@CS NPs, or LED-PLA@CS NPs. The degradation of DNA was excluded as the absorption signal remained stable throughout the control experiment.

### 3.7. Loading Efficiency Experiment

To determine the loading efficiency, free LED was separated from LED@CS NPs and LED-PLA@CS NPs by centrifugation at 12,000 rpm for 30 min at 4 °C. The supernatant containing free drug was collected, and its absorbance was measured at 323 nm using a UV–vis spectrophotometer (PEAK USA model C7000V). The loading efficiency was calculated using Equation (12) [100]. The loading of LED was examined at varying concentrations of LED while maintaining a consistent concentration of CS NPs and PLA@CS NPs. The range of the LED molar concentrations was 10–50 ppm.

(12) $$E\;(\%)=(\frac{A_{o}-A_{t}}{A_{o}})\;\times \;100$$

where *A_o_* is the absorbance of the total amount of drug, and *A_t_* is the absorbance of the free drug.

### 3.8. In Vitro Drug Release

The drug release was evaluated at pH 5.5 to simulate the acidic lysosomal or endosomal environment within cancer cells, which is more acidic than the extracellular tumor microenvironment (pH 6.5–6.8, Section 1). This pH was chosen to mimic the intracellular conditions where nanoparticles are internalized via endocytosis, triggering pH-responsive drug release [17]. Using the dialysis method, the profile of the in vitro drug delivery assays was determined on the manufactured NPs (LED@CS NPs and LED-PLA@CS NPs) in three different releasing media with pH values of 7.4, 5.4, and 5.4, including 10 mM GSH, at 37 °C [101]. In short, 5 mL of LED@CS NPs and LED-PLA@CS NPs solutions (0.015 gm each) were dialyzed against 150 mL of a different PBS solution in dialysis bags (MWCO: 8000–12,000 Da) at 37 °C and 100 rpm. To maintain the volume of the release medium constant, two milliliter aliquots were removed from the dissolution medium at the proper intervals and replaced with the same volume of fresh PBS buffer. A UV–vis spectrophotometer (PerkinElmer, Springfield, IL, USA) was used to measure the amount of released LED in the dialysis bags. It was set to 323 nm, which is the drug’s apparent wavelength. Equation (13) was then utilized to determine the cumulative release (%) [102].

(13) $$\mathrm{Cumulative}\;\mathrm{drug}\;\mathrm{release}\;(\%)=\frac{C_{i}}{C_{total}}\;\times \;100$$

In this case, *C*_t_ represents the drug quantity in the release medium at time *t*, as measured by a UV–vis spectrophotometer, while *C*_total_ denotes the total amount of drug loaded into the nanocarrier.

### 3.9. Drug Release Mechanism

To identify the optimal kinetics model and gain insights into the in vitro drug release mechanism, kinetic regression models, such as first- and second-order kinetic models and the simplified Higuchi model, as well as the Korsmeyer–Peppas model, were used to investigate the release mechanisms of the drug carriers under various environmental conditions. To evaluate the fit and offer information on the release kinetics, the regression equations and the associated r^2^ values were computed.

Our first choice for concentration-dependent drug release is the first-order model governed by Equation (14) [102].

*M*_t_ = *M*_0_ (1 − *e*^*k*1^*t*) (14)

where *k*_1_ is the first-order model′s rate constant, *M*_0_ is the drug′s initial amount, and *M*_t_ is the drug′s amount at time *t*. The second-order model for concentration-dependent drug release is defined by Equation (15) [103].

(15) $$q_{t}=\frac{q_{0}^{2}k_{2}t}{1+q_{e}k_{2}t}$$

where *k*_2_ is the second-order rate constant, *q*_0_ is the drug′s initial amount, and *q_e_* is the drug′s amount at time *t*. A simplified version of the Higuchi model, Equation (16) defines both the drug′s release rate from the polymeric matrix and the drug′s delivery in the pharmaceutical system [102].

(16) $$Q_{t}\;=\;k_{H}\sqrt{t}$$

where *k*_H_ is the kinetic constant according to the defining formulation, and (*Q*_t_) is the cumulative value of the drug release in time *t*. The drug delivery from the carrier systems is expressed using the Korsmeyer–Peppas model [104] using Equation (17).

(17) $$M_{t}/M_{\infty }\;=\;k_{\mathrm{KP}}t^{n}$$

In this pattern, (*M*_t_/*M*_∞_) also displays the cumulative quantity of released medication in time *t*, *k*_KP_ is the kinetic constant of this model, and *n* is the exponent that corresponds to a specific diffusion mechanism.

### 3.10. Antibacterial Assay

Using the agar well diffusion method [105], the antibacterial activity of every compound was assessed against *Salmonella typhi*, *Escherichia coli*, *Staphylococcus aureus*, *Enterococcus faecalis*, *Bacillus subtilis*, and *Klebsiella pneumoniae*. Similar to the disk-diffusion method, this is carried out by uniformly spreading a microbial inoculum across the entire agar surface. Mueller–Hinton agar plates (pH 7.2–7.4 after gelling) without supplementation or standard bacteriology laboratory Mueller–Hinton agar were used in accordance with the CLSI recommendations. First, 100 µL of solutions containing LED, LED@CS NPs, or LED-PLA@CS NPs at a concentration of 50 mg/mL in distilled water were separately added to a 6 mm diameter hole that had been aseptically bored using a sterile cork borer or tip. After that, the inoculated agar plates were incubated in an environment that was appropriate for the test microbe. While incubating, the sample seeped into the microbial strain-seeded agar medium, preventing its growth. At locations where a notable decline in growth was noted, the diameters of the inhibition zones encircling the wells were measured to the closest millimeter. Both quantitative information (inhibition zones in millimeters) and qualitative outcomes (such as classifying microorganisms as susceptible or resistant) can be provided by the agar well diffusion method.

### 3.11. Cell Viability Assay

The cytotoxicity of LED, LED@CS NPs, and LED-PLA@CS NPs was evaluated using methyl thiazolyl tetrazolium (MTT) colorimetry [106] on human lung (WI-38) and liver cancer (HepG2) cell lines. Living cells with an active metabolism can convert MTT into formazan, whereas dying cells lack this capability. Therefore, the MTT test was employed in this investigation to measure the quantity of viable cells. To generate a complete monolayer sheet, the 96-well tissue culture plate was inoculated with 1 × 10^5^ cells per well (100 μL per well) and incubated for 24 h at 37 °C. Following the formation of a confluent cell sheet, the growth medium was carefully removed from the 96-well microtiter plates. The cell monolayer underwent two washes with wash media before the introduction of LED, LED@CS NPs, and LED-PLA@CS NPs. Separate dilutions of LED, LED@CS NPs, and LED-PLA@CS NPs were prepared in RPMI medium with 2% serum (maintenance medium) to achieve final concentrations ranging from 0 to 1000 μg/mL. After that, three wells served as controls, receiving only 100 μL of medium and 10 μL of the MTT stock solution, while 0.1 mL of each dilution was examined in separate wells. The plates were incubated for 24 h at 37 °C. The cells were examined for physical indicators of toxicity, including rounding, shrinkage, cell granulation, and the partial or complete loss of the monolayer. Each well received 20 μL of a 12 mM MTT stock solution (5 mg/mL MTT in sterile PBS) at the conclusion of the incubation period. The MTT was well mixed into the media using a shaking table set to 150 rpm for five minutes. To enable the MTT to be metabolized, the plate was then incubated for four hours at 37 °C with 5% CO_2_. After removing the MTT solution, 200 μL of DMSO was added to the purple formazan (MTT metabolic product) crystal that had formed at the bottom of the wells. The formazan was completely mixed with the solvent by spinning the shaking table at 150 rpm for five minutes. An ELISA reader (StatFax-2100, Awareness Technology Inc., Palm City, FL, USA) was used to measure the optical density at 570 nm. Equation (18) was utilized to ascertain the percentage of cell inhibition.

*% Cell Inhibition* = 100 − 100 × (*T* − *T*_0_)/(*C* − *T*_0_) (18)

where *T* is the test sample′s optical density, the optical density at time zero is denoted as *T*_0_, and the optical density of the control is denoted as *C*. The number of cells and optical density should be proportional.

## 4. Conclusions

This study successfully developed dual-responsive chitosan–polylactic acid nanosystems (PLA@CS NPs) for targeted ledipasvir (LED) delivery to HepG2 liver cancer cells, demonstrating enhanced therapeutic selectivity and reduced systemic toxicity. Two formulations—LED@CS NPs (143 nm, +43.5 mV) and LED-PLA@CS NPs (394 nm, +33.3 mV)—achieved high drug-loading capacities (44.1% and 89.3%, respectively), with the latter showing superior payload efficiency. Since most drugs work through interaction with DNA, the in vitro affinity of DNA to LED and its encapsulated forms were assessed using stopped-flow and other approaches. They bind through multi-modal electrostatic and intercalative modes via two reversible processes: a fast complexation followed by a slow isomerization. The overall binding activation parameters for LED (*K*_a_ = 128.4 M^−1^, *K*_d_ = 7.8 × 10^−3^ M, Δ*G* = −12.02 kJ mol^−1^), LED@CS NPs (*K*_a_ = 2131 M^−1^, *K*_d_ = 0.47× 10^−3^ M, Δ*G* = −18.98 kJ mol^−1^) and LED-PLA@CS NPs (*K*_a_ = 22026 M^−1^, *K*_d_ = 0.045 × 10^−3^ M, Δ*G* = −24.79 kJ mol^−1^) were obtained with a reactivity ratio of 1/16/170 (LED/LED@CS NPs/LED-PLA@CS NPs). This indicates that encapsulation enhanced the interaction between the DNA and the LED-loaded nanoparticle systems without changing the mechanism and formed thermodynamically stable complexes. Under tumor-mimetic conditions (pH 5.5, 10 mM GSH), both systems demonstrated controlled, stimuli-responsive release, achieving 88% for LED@CS NPs and 73% for LED-PLA@CS NPs over a period of 50 h, which surpassed the performance under physiological conditions (58% and 54%). The dual responsiveness to pH and redox conditions, regulated by Fickian diffusion, facilitated accurate tumor-targeted delivery. Although encapsulation did not improve the immediate cytotoxicity relative to free LED, as shown by an in vitro cell-survival study on HepG2 cancer cell lines, it significantly increased the therapeutic index (2.1-fold for LED-PLA@CS NPs) by protecting non-cancerous cells. Additionally, antibacterial assessments were performed on six bacterial strains, revealing that LED-PLA@CS NPs exhibited greater antibacterial activity compared to LED and LED@CS NPs, which aligns with the kinetic results, highlighting their potential for preventing chemotherapy-associated infections. By enabling controlled LED release with minimized off-target effects, PLA@CS NPs represent a promising strategy for repurposing antiviral drugs into safer, more effective cancer nanomedicines. Future development of LED@CS NPs and LED-PLA@CS NPs for HCV or cancer treatment could explore oral or intravenous administration. Chitosan’s mucoadhesive properties (Section 1) support oral delivery, improving the bioavailability [24], while the nanoparticles’ size (143–394 nm) suits intravenous EPR targeting to the liver. Preclinical studies will assess the pharmacokinetics and efficacy in vivo, alongside EPR validation and active targeting strategies like ligand conjugation. These findings support LED-based nanotherapeutics for clinical translation.

## Figures and Tables

**Table 1:** Mean size, polydispersity index (PDI) and zeta potential of LED, LED@CS NPs and LED-PLA@CS NPs.

	LED	LED@CS NPs	LED-PLA@CS NPs
Mean Size	152.4 nm	142.5 nm	393.7
PDI	0.277	0.304	0.389
Zeta Potential	+9.44 mV	+43.51 mV	+33.27

**Table 2:** *T*_m_ of free DNA, DNA-LED, DNA-LED@CS NPs, and DNA-LED-PLA@CS NPs complexes.

	*T*_m_
DNA free	72.1
DNA-LED complex	71.8
DNA-LED@CS NPs complex	71.3
DNA-LED-PLA@CS NPs complex	75.2

**Table 3:** Activation parameters for the first step of DNA binding to LED, LED@CS NPs, and LED-PLA@CS NPs.

	LED	LED@CS NPs	LED-PLA@CS NPs
*k*_1_ [M^−1^ s^−1^]	2.5 ± 0.3	5.72 ± 0.60	18.2 ± 2.2
*k*_−1_ [s^−1^]	0.56 ± 0.01	0.22 ± 0.02	0.15 ± 0.07
*K*_a1_ [M^−1^]	4.48	26.0	120
*K*_d1_ [10^−2^ M]	22.0	3.8	0.83
*ΔG*_1_ [kJ mol^−1^]	−3.72	−8.07	−11.85

**Table 4:** Activation parameters for the second step of DNA binding to LED, LED@CS NPs, and LED-PLA@CS NPs.

	LED	LED@CS NPs	LED-PLA@CS NPs
*k*_2_ [M^−1^ s^−1^]	1.63 ± 0.58	0.24 ± 0.02	3.58 ± 0.3
*k*_−2_ [10^−2^ s^−1^]	6.0 ± 1.0	0.3 ± 0.1	2.0 ± 0.7
*K*_a2_ [M^−1^]	27.2	80.0	179.0
*K*_d2_ [10^−2^ M]	3.67	1.25	0.55
*ΔG*_2_ [kJ mol^−1^]	−8.14	−10.81	−12.80

**Table 5:** Activation parameters for the overall reaction of DNA binding to LED, LED@CS NPs, and LED-PLA@CS NPs.

	LED	LED@CS NPs	LED-PLA@CS NPs
*K*_d_ [10^−3^ M]	7.8	0.47	0.045
*K*_a_ [M]	128.4	2131	22,026
*ΔG* [kJ mol^−1^]	−12.02	−18.98	−24.79

**Table 6:** Release kinetic analysis of LED from LED@CS NPs and LED−PLA@CS NPs formulation in different pH media.

Kinetic Model	LED@CS NPs			LED-PLA@CS NPs		
	pH = 7.4	pH = 5.4	pH = 5.4 + 10 mM GSH	pH = 7.4	pH = 5.4	pH = 5.4 + 10 mM GSH
First order	0.989	0.944	0.935	0.739	0.784	0.814
Second order	0.941	0.978	0.973	0.866	0.882	0.900
Higuchi	0.977	0.963	0.943	0.961	0.968	0.940
**Korsmeyer–Peppas**	**0.979**	**0.987**	**0.978**	**0.992**	**0.993**	**0.981**

**Table 7:** Kinetic constant (*k*) and release exponent (*n*) for LED release from LED–encapsulated LED@CS NPs and LED–PLA@CS NPs using the Korsmeyer–Peppas equation at 37 °C.

	LED@CS NPs		LED-PLA@CS NPs	
	*n*	*K*_kP_	*n*	*K*_kP_
pH = 7.4	0.37	0.14	0.22	0.24
pH = 5.4	0.42	0.18	0.23	0.26
pH = 5.4 + 10 mM GSH	0.48	0.24	0.25	0.29

**Table 8:** IC_50_ and therapeutic index of LED in the free form and encapsulated forms on HepG2 cancer cells and WI-38 normal cells using the MTT assay.

Samples	IC_50_ (μg ml^−1^)		Therapeutic Index (TI)
	HepG2 Cancer Cells	WI-38 Cells	
LED	101.76 ± 0.33	184.46 ± 2.67	1.81
LED@CS NPs	110.46 ± 1.35	232.85 ± 2.63	2.11
LED-PLA@CS NPs	144.76 ± 0.69	341.77 ± 3.32	2.36

## Data Availability

The data that support the findings of this study are available within the article and its Supplementary Materials.

## Supplementary Materials

The following supporting information can be downloaded at https://www.mdpi.com/article/10.3390/ijms26136070/s1.
