# Supplementary material for: GSH/pH-Responsive Chitosan–PLA Hybrid Nanosystems for Targeted Ledipasvir Delivery to HepG2 Cells: Controlled Release, Improved Selectivity, DNA Interaction, Electrochemical and Stopped-Flow Kinetics Analyses
Source: Int J Mol Sci. 2025 Jun 24;26(13):6070. doi: 10.3390/ijms26136070 (PMC12250219; doi:10.3390/ijms26136070)
Supplement: Supplementary file 1 [file ijms-26-06070-s001.zip › ijms-3714326-supplementary.pdf]

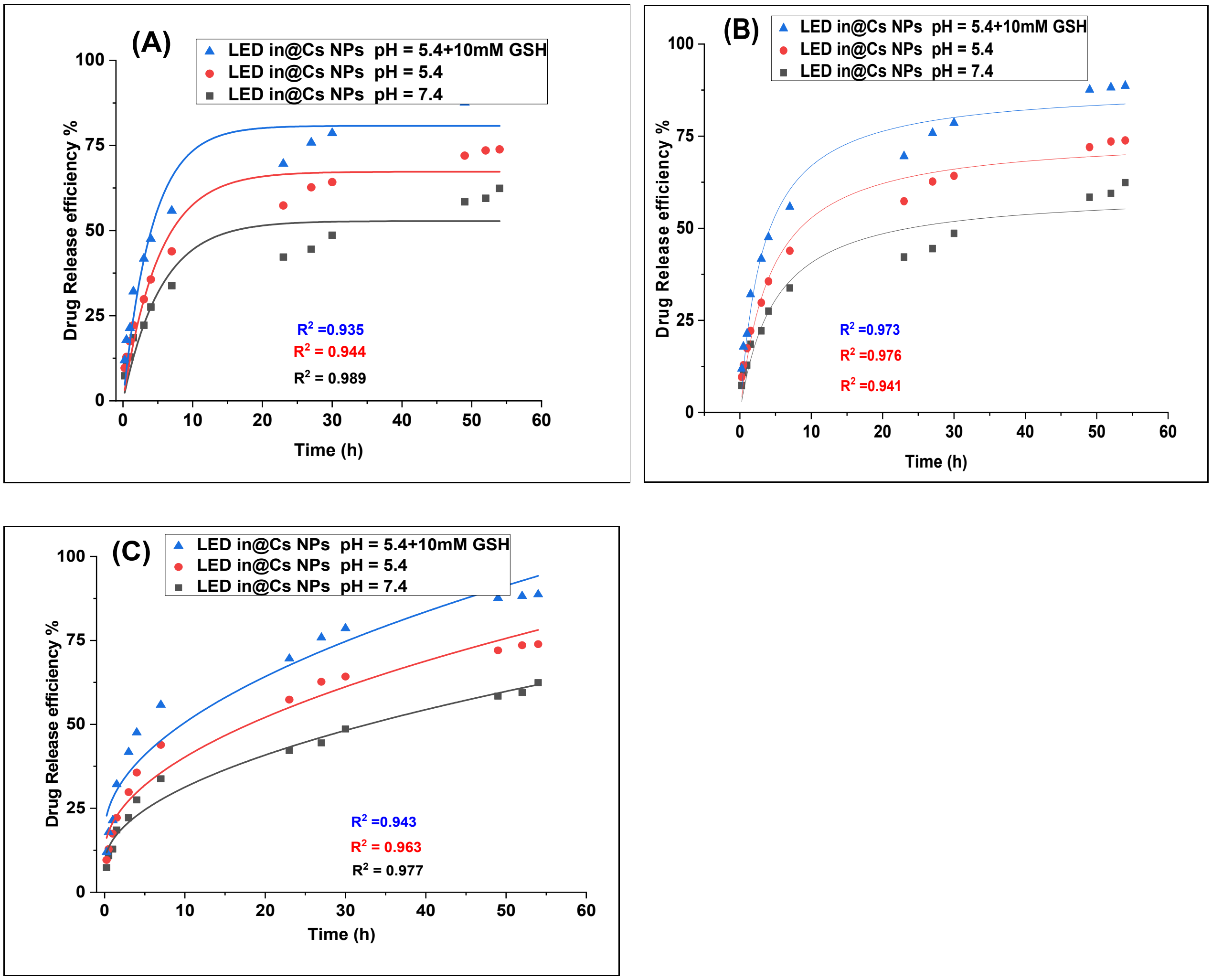

Figure S1. LED release fitting curves from LED in@ CS NPs by (A) first-order kinetics, (B) second-order kinetics, and (C) Higuchi kinetics.

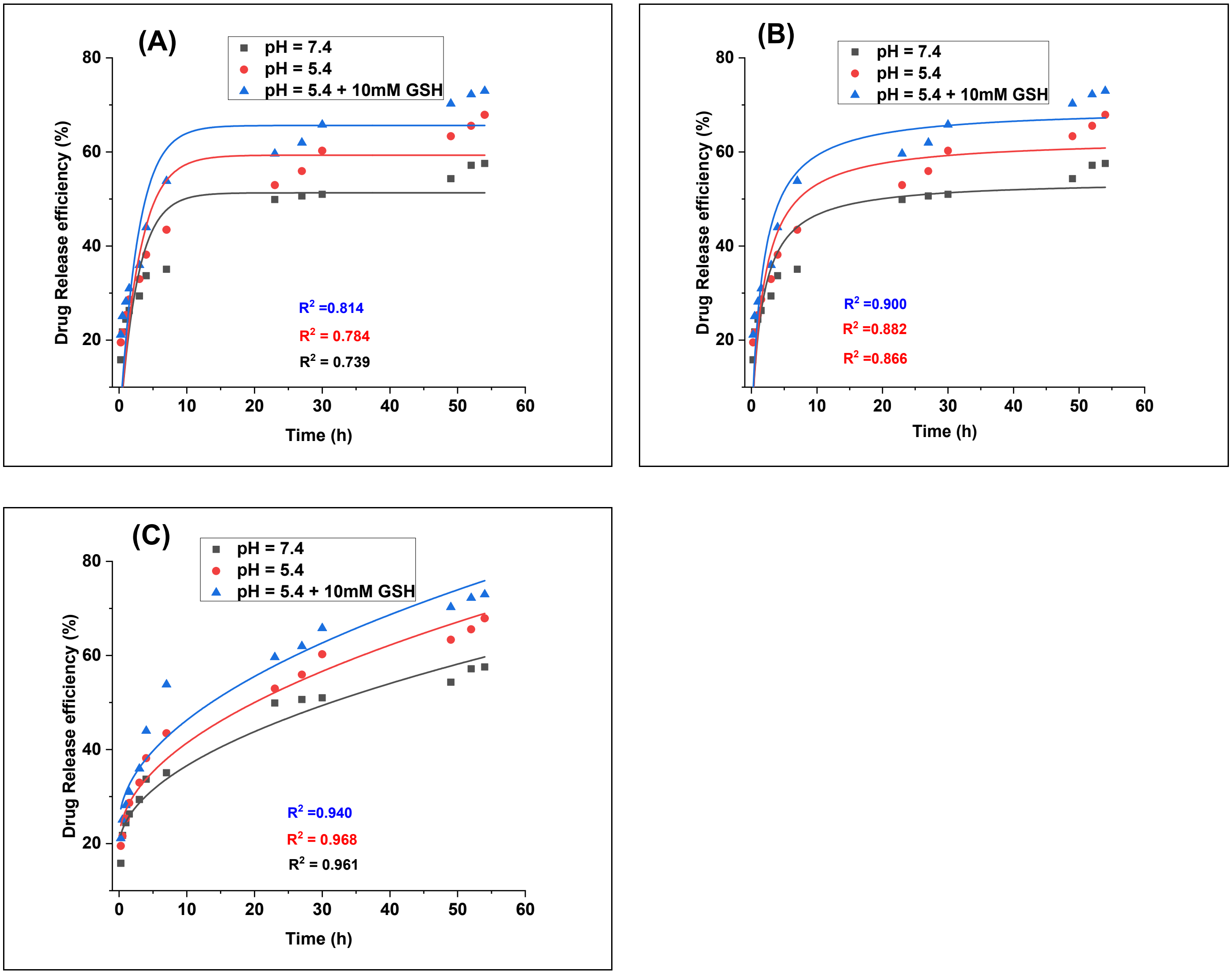

Figure S2. LED release fitting curves from PLA-LED@CS NPs by (A) first-order kinetics, (B) second-order kinetics, and (C) Higuchi kineticsl.
